# Impact of MEK Inhibition on Childhood RASopathy-Associated Hypertrophic Cardiomyopathy

**DOI:** 10.1016/j.jacbts.2024.10.002

**Published:** 2024-12-04

**Authors:** Cordula M. Wolf, Martin Zenker, Olga Boleti, Gabrielle Norrish, Mark Russell, Joshua K. Meisner, David M. Peng, Terence Prendiville, Jake Kleinmahon, Paul F. Kantor, Danielle Gottlieb Sen, Derek G. Human, Peter Ewert, Marcus Krueger, Daniela Reber, Birgit Donner, Christopher Hart, Irena Odri Komazec, Stefan Rupp, Andreas Hahn, Anja Hanser, Michael Hofbeck, Jos M.T. Draaisma, Floris E.A. Udink ten Cate, Alessandro Mussa, Giovanni B. Ferrero, Laurence Vaujois, Marie-Josée Raboisson, Marie-Ange Delrue, Christopher Marquis, Yves Théoret, Soujanya Bogarapu, Adrian Dancea, Mette Moller Handrup, Mariska Kemna, Tiina Ojala, Niti Dham, Frank Dicke, Tim Friede, Juan Pablo Kaski, Bruce D. Gelb, Gregor Andelfinger

**Affiliations:** a Department of Congenital Heart Defects and Pediatric Cardiology, German Heart Center Munich, School of Medicine and Health, Technical University of Munich, Munich, Germany; b Deutsches Zentrum für Herz-Kreislauf-Forschung (German Center for Cardiovascular Research), partner site Munich Heart Alliance, Munich, Germany; c Member of the European Reference Network for Rare and Low Prevalence Complex Diseases of the Heart; d Institute of Human Genetics, University Hospital Magdeburg, Otto-von-Guericke-University, Magdeburg, Germany; e Centre for Inherited Cardiovascular Diseases, Institute of Cardiovascular Science, University College London and Great Ormond Street Hospital, London, United Kingdom; f University of Michigan, Ann Arbor, Michigan, USA; g Children’s Health Ireland at Crumlin, Crumlin, Ireland; h Ochsner Hospital for Children, Jefferson, Louisiana, USA; i Children’s Hospital Los Angeles, Los Angeles, California, USA; j Johns Hopkins School of Medicine, Baltimore, Maryland, USA; k British Columbia’s Children’s Hospital, Vancouver, British Columbia, Canada; l Department of Neonatology, Municipal Hospital Munich Schwabing, Munich, Germany; m Pediatric Cardiology, University Children’s Hospital of Basel, University of Basel, Basel, Switzerland; n Department of Pediatric Cardiology, Pediatric Heart Center, Children’s Hospital, University of Bonn, Bonn, Germany; o Department of Child and Adolescent Health (Pediatrics III, Pediatric Cardiology), Medical University of Innsbruck, Innsbruck, Austria; p Pediatric Heart Center, University of Giessen and Marburg, Giessen, Germany; q Department of Child Neurology, University of Giessen, Giessen, Germany; r Department of Pediatric Cardiology and Intensive Medicine, University Hospital Tübingen, Eberhard-Karls-University, Tübingen, Germany; s Radboud University Medical Center, Radboud Institute for Health Sciences, Amalia Children’s Hospital, Department of Pediatrics, Nijmegen, the Netherlands; t Department of Public Health and Pediatric Sciences, University of Turin, Turin, Italy; u Department of Clinical and Biological Sciences, School of Medicine, University of Turin, Turin, Italy; v Centre Mère-Enfant Soleil, Université de Laval, Quebec City, Quebec, Canada; w CHU Sainte Justine, Department of Pediatrics, Université de Montréal, Montréal, Quebec, Canada; x Children’s Hospital of Illinois, University of Illinois College of Medicine, Chicago, Illinois, USA; y Montreal Children’s Hospital, McGill University Health Center, Montréal, Quebec, Canada; z Center for Rare Diseases, Aarhus University Hospital, Copenhagen, Denmark; aa Seattle Children’s Hospital, Seattle, Washington, USA; bb New Children’s Hospital Pediatric Research Center, Helsinki University Hospital, Helsinki, Finland; cc George Washington University and Children’s National Hospital, Washington, District of Columbia, USA; dd Alberta Children’s Hospital, University of Calgary, Calgary, Alberta, Canada; ee University Medical Center Göttingen, Department of Medical Statistics, Göttingen, Germany; ff Deutsches Zentrum für Herz-Kreislauf-Forschung (German Center for Cardiovascular Research), partner site Lower Saxony, Göttingen, Germany; gg Deutsches Zentrum für Kinder- und Jugendgesundheit (German Center for Child and Adolescent Health), partner site Göttingen, Göttingen, Germany; hh Mindich Child Health and Development Institute and Departments of Pediatrics and Genetics and Genomic Sciences, Icahn School of Medicine at Mount Sinai, New York, New York, USA

**Keywords:** childhood cardiomyopathy, MEK inhibition, Noonan syndrome spectrum, RAS/MAPK signaling, RASopathies, survival

## Abstract

There is an unmet medical need to treat patients with severe hypertrophic cardiomyopathy leading to heart failure and death in children carrying pathogenic activating variants in the RAS/mitogen-activated protein kinase pathway. A retrospective analysis of 61 patients provides evidence for decreased mortality and morbidity with improved cardiac status in patients with RASopathy with severe hypertrophic cardiomyopathy receiving mitogen-activated protein kinase kinase inhibition (n = 30) vs those with standard-of-care treatment (n = 31). Side effects were not life threatening and were manageable. The data presented suggest that personalized therapies targeting underlying signaling pathway abnormalities might be effective in critically ill patients with RASopathy warranting clinical investigation.

## Highlights

• RAS/MAPK variants cause infant hypertrophic cardiomyopathy, increasing morbidity.
• Hyperactivated pathway inhibition improves cardiomyopathy in RASopathy mouse models.
• Retrospective study: 30 children on trametinib vs 31 on standard care for cardiomyopathy.
• Trametinib significantly reduced death, transplant, and cardiac surgery risk.
• Trametinib showed no severe adverse events; frequent skin and mucous side effects noted.

## Visual Abstract

**Figure:**
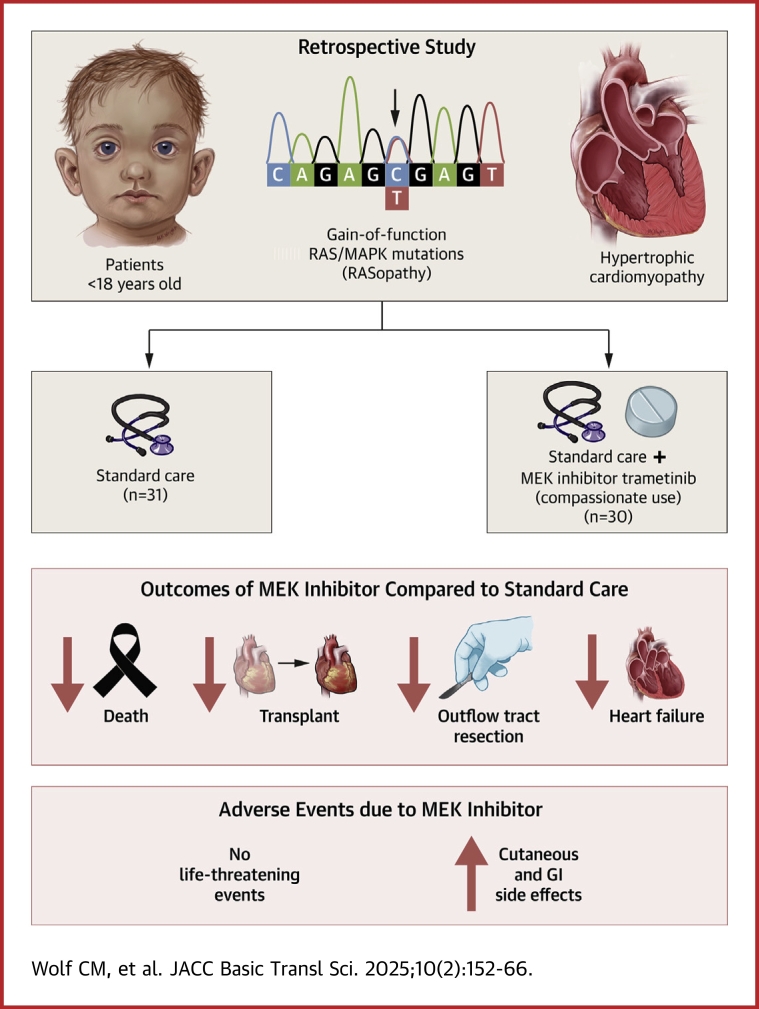

RASopathy-associated hypertrophic cardiomyopathy (RAS-HCM) presenting during the first year of life carries a poor prognosis, with considerable mortality.^1^^,^^2^ RASopathies are caused by pathogenic variants in genes encoding for transducers of the RAS/mitogen-activated protein kinase (MAPK) signal cascade and are characterized by abnormalities of multiple organ systems.^3^ This group of developmental diseases includes Noonan syndrome (NS), the most common entity, with an estimated incidence of about 1 in 2,500;^4^ NS with multiple lentigines (NSML); Costello syndrome; and cardiofaciocutaneous syndrome.

Roughly 20% of patients with RASopathies have a hypertrophic cardiomyopathy.^5^^,^^6^ On the basis of data from as far back as the 1980s, infants <6 months of age who present with RAS-HCM and heart failure have a mortality rate of approximately 60% in the first year of life, usually attributable to progressive heart failure.^2^^,^^7^ Concomitant ventricular outflow tract obstruction and congenital heart defects, such as pulmonary valve stenosis and atrial septal defects, additionally contribute to morbidity in those children.^5^^,^^8^^,^^9^ RAS-HCM also is an underreported indication for cardiac transplantation, with significant procedure-related mortality.^10^ The clinical course can be further complicated by lymphatic dysplasia, pulmonary lymphangiectasia, pulmonary hypertension, feeding problems, renal abnormalities, and other issues.^11^

The majority of patients with RAS-HCM have a diagnosis of NS and carry gain-of-function missense variants of *RAF1*, *RIT1*, and other genes that drive RAS/MAPK signaling through hyperactivation of ERK1/2.^12^ In patients with NSML, which is commonly associated with RAS-HCM, the causal *PTPN11* variants produce phosphatase-impaired mutant proteins that lead to incompletely defined, probably neomorphic effects on MAPK signaling and also increased AKT/mTOR activity.^13^ Preclinical studies in transgenic mice with an NS-associated *Raf1* variant recapitulated RASopathy features, including myocardial hypertrophy, and exhibited increased signaling through the RAS/MAPK pathways.^14^^,^^15^ Pharmacologic treatment with a MAPK kinase (MEK)^14^^,^^15^ inhibitor in NS mice was beneficial for both cardiac and extracardiac manifestations.

We reported the first use of MEK inhibition (MEKi) in 2 patients with NS due to pathogenic *RIT1* variants, resulting in amelioration of heart failure.^16^^,^^17^ In the 2 patients, decrease in ventricular mass and reduction in subvalvular and valvular stenosis occurred.^16^ Since then, improvement of cardiac status has been reported in an additional 8 patients treated with MEKi for severe RAS-HCM.^18^, ^19^, ^20^, ^21^

The goal of this retrospective analysis was to evaluate the effect of compassionate or off-label use of MEKi in addition to standard of care in a cohort of 61 children with RAS-HCM admitted to the hospital for heart failure and/or cardiac surgery for outflow tract resection. Multivariable analysis and propensity scoring for adjustment suggest that genotype-dependent MEKi treatment has the potential to improve survival in critically ill children with RAS-HCM and to serve as alternative to cardiac surgery if indicated in patients with RAS-HCM, providing impetus for prospective clinical trials.

## Methods

### Study population, setting, and data collection

A retrospective cohort analysis was carried out at 23 tertiary care centers specializing in congenital heart disease and pediatric cardiology from the United States, Canada, Austria, Denmark, Finland, Germany, Ireland, Italy, the Netherlands, and the United Kingdom. Clinical data were collected from medical records from all patients meeting the following inclusion criteria: 1) genetic diagnosis of a RASopathy due to a (likely) pathogenic gain-of-function variant in an established RASopathy gene;^22^ 2) echocardiographic diagnosis of hypertrophic cardiomyopathy; and 3) admission to the hospital since 2000 with either heart failure and/or severe outflow tract obstruction warranting intervention.

Patients carrying genetic variants of the *PTPN11* gene were excluded from this analysis because the pathogenic mechanism for hypertrophy is not mediated primarily through increased MAPK signaling.^13^^,^^23^

Patients who received compassionate or off-label MEKi in addition to standard therapies are designated as the “MEKi group,” and those receiving standard therapies only as “control subjects.” Standard therapies were defined as current standard-of-care conservative and interventional treatment strategies as initiated by the treating physicians, such as beta-blockers, calcium-channel blockers, diuretic agents, inotropes, and cardiac interventions.

The treatment group included 5 previously reported patients,^16^^,^^18^^,^^20^^,^^21^ for whom we now report updated and long-term follow-up. Part of the control cohort was previously published.^1^^,^^9^^,^^24^

The primary endpoint was a composite of cardiac surgery for outflow tract resection, heart transplantation or death. The secondary endpoints were: 1) cardiac surgery for outflow tract resection; 2) death or heart transplantation; and 3) death or heart transplantation in the subgroup of critically ill patients presenting in severe heart failure (Ross class IV; n = 16).

Clinical variables collected by standardized questionnaire included weight; height; comorbidities; other structural heart defects; number of cardiac medications; cardiac interventions and surgical procedures; transthoracic echocardiographic variables of peak outflow tract gradient, maximal end-diastolic myocardial wall thickness, left ventricular end-diastolic diameter, and left ventricular mass indexed to body surface area; and the laboratory heart failure markers N-terminal pro–brain natriuretic peptide (NT-proBNP) or brain natriuretic peptide. Data on arrhythmias were collected, and arrhythmias were defined as severe when requiring medical intervention, resuscitation, or defibrillation. Variables were obtained at hospital admission (baseline time point) and at the last follow-up time point available, with follow-up time administratively censored at 36 months after baseline. Additionally, Ross classification, laboratory heart failure biomarkers, and individual echocardiographic parameters of cardiomyopathy (peak outflow tract gradient, maximal end-diastolic wall thickness *z*-score, left ventricular end-diastolic diameter, and left ventricular mass indexed to body surface area) were analyzed at 1, 3, 6, and 12 months after baseline, when available, in the MEKi group, with patients experiencing events censored. Where applicable, data were related to normal values and *z*-scores.^25^, ^26^, ^27^, ^28^

The severity of heart failure was expressed according to the classification for infants proposed by Ross et al^29^ (class I, no signs; class II, mild; class III, moderate; and class IV, severe). NT-proBNP or brain natriuretic peptide values were assessed as a heart failure biomarker.^30^, ^31^, ^32^

Consideration for cardiac surgery for outflow tract resection in the treatment group was defined according to criteria defined in the 2014 European Society of Cardiology guidelines^33^ (outflow tract gradient ≥50 mm Hg plus Ross class ≥III) or by the presence of severe left ventricular outflow tract gradient (≥90 mm Hg) independent of Ross classification. This also applied to all patients of the control group admitted to the hospital prior to 2014.

### Treatment regimen

All treating physicians followed local guidelines for off-label or compassionate drug use, approved by the local ethical committees or Institutional Review Boards. Only deidentified data were used for analysis.

### Statistical analysis

Descriptive statistics were used to summarize the data. Results are reported as median (Q1-Q3) for continuous variables and as counts and percentages for categorical variables.

Follow-up time interval was administratively censored at 36 months in all statistical analysis with the exception of the analysis of clinical outcome during follow-up in the MEKi group, which was performed only for the first 12 months of follow-up given the large number of missing data beyond this timepoint.

The main statistical analysis included the comparison between the MEKi group and control subjects.

A subgroup analysis was performed for only patients who presented in severe heart failure, as defined by Ross class IV, and comparing the MEKi group and control subjects among those patients.

Comparisons between groups were assessed using chi-square or Fisher exact tests for categorical variables and the Mann-Whitney *U* test for quantitative variables.

Time-to-event endpoints are displayed as Kaplan-Meier curves. For the secondary endpoint of cardiac surgery for outflow tract resection, cumulative incidence functions (Aalen-Johansen estimators) with death and heart transplantation as competing events are shown. Unadjusted differences between groups are reported as HRs with 95% CIs and were compared using the log-rank test. In competing event analyses, Gray’s test was applied. In addition, several analyses adjusted for possible confounders were conducted. Following recommendations for small nonrandomized studies,^34^ these included Cox proportional hazards regression including confounders as covariates and a propensity score as covariate. The proportionality assumption of the Cox model was checked visually using log-minus-log plots of the survival probabilities. As confounders, the following variables were considered: sex, age at hospital admission, prematurity, Ross classification at hospital admission, and prior cardiac surgery for outflow tract obstruction. These were included in the multivariable regression model and also in the propensity score. The latter was included as a linear predictor in the regression model.

In the Ross class IV subgroup analysis, the Firth correction was applied in the calculation of the propensity scores. The same confounders described earlier were considered.

In the MEKi cohort, repeated-measures analyses of the biomarker NT-proBNP and echocardiographic variables (including peak outflow tract gradient, maximal end-diastolic myocardial wall thickness *z*-score, left ventricular end-diastolic diameter *z*-score, and left ventricular mass indexed to body surface area) were carried out. The linear models include intercepts (baseline) and changes over time (1, 3, 6, and 12 months of follow-up). An autoregressive correlation structure between observations from the same subject is assumed. Least squares means with SEs are reported.

Because of the exploratory nature of the analyses, *P* values were not adjusted for multiple testing. Two-sided *P* values ≤0.05 were considered to indicate statistical significance. Analyses were performed using SPSS Statistics version 28 (IBM) and SAS version 9.4 (SAS Institute).

## Results

### Cohort characteristics at baseline time point

Data from 61 patients (30 male patients [49%], median age 7.4 months [Q1-Q3: 1.8-35.6 months]) admitted to the hospital between 2000 and 2023 and meeting inclusion criteria were analyzed. Demographic and clinical characteristics, including molecular genetic diagnoses, comorbidities, other structural heart defects, and echocardiographic variables at hospital admission, are summarized in Table 1. Most patients (66%) were admitted within their first 12 months of life, with the most common diagnosis being NS (90%) with an underlying pathogenic variant in the *RAF1* gene (66%). The most common reason for hospital admission was heart failure (Ross classes III and IV) with outflow tract obstruction in 48% of patients and severe outflow tract obstruction (outflow tract gradient of 90 mm Hg or higher) without heart failure in 38% of patients. There was severe left ventricular hypertrophy on echocardiography (maximal end-diastolic left ventricular wall thickness *z*-score) (median 4.5; Q1-Q3: 3.9-5.2; range: 2.6-9.6). The main comorbidities included prematurity, respiratory failure, and lymphatic dysplasia, occurring in 28%, 18%, and 20% of patients, respectively.

**Table 1:** Patient Characteristics and Clinical Features at Baseline

	Total (N = 61)	Standard of Care Plus MEKi Group (n = 30)	Standard of Care Only Group (Control) (n = 31)	*P* Value^a^
Age of RAS-HCM diagnosis, mo	0.5 (0.0-2.3) [0-132]	1.0 (0.0-2.0) [0-48]	0.25 (0.0-3.0) [0-132]	0.59
Age at hospital admission, mo	7.4 (1.8-35.6) [0-214]	5.0 (1.9-34.3) [0-214]	11.0 (1.1-37.0) [0-180]	0.66
Age category at hospital admission				0.33
0-6 mo	29 (48)	16 (53)	13 (42)	
> 6-12 mo	11 (18)	4 (13)	7 (23)	
>12-24 mo	2 (3)	2 (7)	0	
>2-6 y	7 (12)	2 (7)	5 (16)	
>6-12 y	6 (10)	4 (13)	2 (7)	
>12-18 y	6 (10)	2 (7)	4 (13)	
Year at hospital admission	2000-2023	2017-2022	2000-2023	
Male	30 (49)	14 (47)	16 (52)	0.80
Type of RASopathy				0.34
Noonan syndrome	55 (90)	26 (87)	29 (94)	
Cardiofaciocutaneous syndrome	2 (3)	2 (7)	0	
Costello syndrome	4 (7)	2 (7)	2 (7)	
Causal gene				0.25
*RAF1*	40 (66)	20 (67)	20 (66)	
*RIT1*	12 (20)	5 (17)	7 (23)	
*LZTR1*	3 (5)	1 (3)	2 (7)	
*BRAF*	2 (3)	2 (7)	0	
*HRAS*	2 (3)	2 (7)	0	
*KRAS*	2 (3)	0	2 (7)	
Comorbidities				
Prematurity	17 (28)	10 (33)	7 (23)	0.40
Respiratory failure requiring ventilation	11 (18)	6 (20)	5 (16)	0.75
Extracorporeal membrane oxygenation	1 (2)	1 (3)	0	0.49
Lymphatic dysplasia	12 (20)	7 (23)	5 (16)	0.53
Pulmonary hypertension	6 (10)	3 (10)	3 (10)	1.0
Severe arrhythmia (requiring medication or defibrillation/resuscitation)	6 (10)	4 (13)	2 (7)	0.34
Other structural heart defect				
Dysplastic pulmonary valve	21 (34)	12 (40)	9 (29)	0.43
Secundum type atrial septal defect	20 (33)	10 (33)	10 (32)	1.0
Dysplastic mitral valve	13 (21)	7 (23)	6 (19)	0.76
Dysplastic aortic valve	6 (10)	4 (13)	2 (7)	0.43
Ventricular septal defect	5 (8)	2 (7)	3 (10)	1.0
Coarctation of the aorta	3 (5)	2 (7)	1 (3)	0.61
Invasive procedures prior to hospital admission				0.63
None	44 (72)	20 (67)	24 (77)	
Cardiac interventional catheterization	6 (10)	3 (10)	3 (10)	
Cardiac surgery	10 (16)	6 (20)	4 (13)	
Cardiac interventional catheterization and cardiac surgery	1 (2)	1 (3)	0	
Past cardiac intervention^b^				
Balloon valvuloplasty	3 (5)	2 (7)	1 (3)	0.61
LVOTO or RVOTO resection	6 (10)	6 (20)	0	0.01
Mitral valve plasty	1 (2)	1 (3)	0	0.49
Reason for hospital admission				0.92
Heart failure (Ross class ≥III) without outflow tract obstruction	9 (15)	4 (13)	5 (16)	
Heart failure (Ross class ≥III) with outflow tract obstruction	29 (48)	14 (47)	15 (48)	
Severe outflow tract obstruction without heart failure (Ross class <III)	23 (38)	12 (40)	11 (36)	
Ross classification	3 (2-4) [1-4] (n = 61)	3 (2-4) [1-4] (n = 30)	3 (2-3) [1-4] (n = 31)	0.96
Ross class				0.23
I	7 (12)	5 (17)	2 (7)	
II	18 (30)	8 (27)	10 (32)	
III	20 (33)	7 (23)	13 (42)	
IV	16 (26)	10 (33)	6 (20)	
Cardiac medications	2 (1-3) [0-6] (n = 59)	2 (1-3) [0-5] (n = 30)	2 (1-3) [0-6] (n = 29)	0.98
Echocardiographic variables				
Myocardial wall thickness *z*-score^c^	4.5 (3.9-5.2) [2.6-9.6] (n = 53)	4.4 (3.8-5.2) [2.6-9.3] (n = 28)	4.6 (4.0-5.7) [3.0-5.6] (n = 25)	0.46
Peak outflow tract gradient,^d^ mm Hg	70 (52-92) [2-211] (n = 59)	67 (54-89) [5-211] (n = 30)	70 (44-100) [2-143] (n = 29)	0.69
Outflow tract obstruction^e^				0.64
None	7 (12)	2 (7)	5 (16)	
Both sides	16 (26)	9 (30)	7 (23)	
Predominant LVOTO	31 (51)	15 (50)	16 (52)	
Predominant RVOTO	7 (12)	4 (13)	3 (10)	

Values are median (Q1-Q3) [range] or n (%). Percentages may not total 100 because of rounding.

LVOTO = left ventricular outflow tract obstruction; MEKi = mitogen-activated protein kinase kinase inhibition; RAS-HCM = RASopathy-associated hypertrophic cardiomyopathy; RVOTO = right ventricular outflow tract obstruction.

a Pearson chi-square or Fisher exact test for categorical variables and nonparametric Mann-Whitney test for independent continuous variables.

b Prior cardiac surgery occurred at >12 weeks prior to baseline time point.

c Maximal myocardial wall thickness *z*-score of the end-diastolic interventricular septum or the end-diastolic left ventricular posterior wall.

d Peak gradient over left or right outflow tract.

e Defined as dp >30 mm Hg.

Pulmonary valve stenosis and secundum-type atrial septal defect were the most common concomitant cardiac defects (34% and 33% of patients, respectively).

Thirty-one patients (51%) received standard therapy only; 30 patients (49%) received additional compassionate-use or off-label MEKi.

Patient characteristics were comparable between the treatment and control groups in all variables, with the exception of significantly more patients in the MEKi group having undergone prior surgical outflow tract intervention before hospital admission and the time of admission to the hospital varying between 2000 and 2023 in the control and between 2017 and 2022 in the MEKi group (Supplemental Figure 1).

All patients in the control group admitted for surgical intervention met the 2014 criteria for consideration of surgical intervention according to their Ross classification and outflow tract gradients, regardless of their year of hospital admission. All patients in the MEKi group admitted for surgical intervention met the 2014 criteria for consideration of surgical intervention according to their Ross classification and outflow tract gradients, regardless of having undergone prior surgical outflow tract resection.

In era analysis, there was no difference in reaching the primary composite endpoint comparing patients in the standard-of-care group only meeting inclusion criteria from 2000 until 2010, 2011 until 2015, and 2016 until 2023 (Supplemental Figure 2).

### Clinical outcome during follow-up

Median observation time until death, transplantation, or last clinical follow-up was 18 months (Q1-Q3: 8.6-36 months; range: 0.25-36 months) and was administratively censored at 36 months. The total follow-up in the MEKi cohort was 555 patient-months, and in the control cohort 706 patient-months.

**Figure 1:**
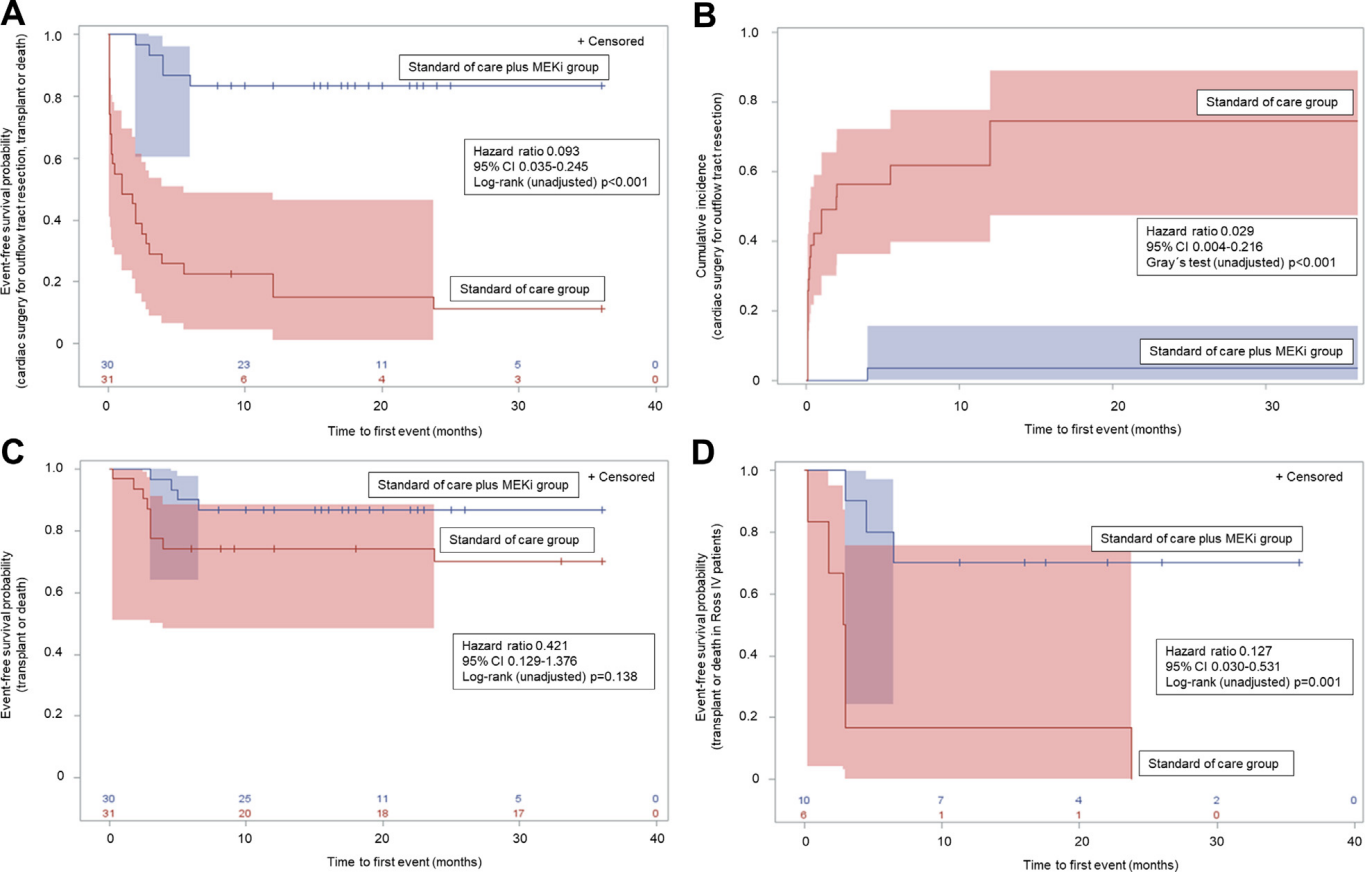
Clinical Outcomes During Follow-Up of Retrospective Cohort Analysis: Kaplan-Meier Curves for Primary and Secondary Endpoints (A) Time-to-event endpoints displayed as Kaplan-Meier curve for the primary composite endpoint of cardiac surgery for outflow tract obstruction or heart transplantation or death; product-limit survival estimates with number of subjects at risk and 95% Hall-Wellner bands; differences between groups are reported as HRs with 95% CIs and were tested using log-rank tests. Time 0 reflects the baseline time point when meeting inclusion criteria and admission to hospital. (B) Cumulative incidence function (Aalen-Johansen estimator) with 95% confidence limits for the secondary endpoint of cardiac surgery for outflow tract resection with death and heart transplantation as competing events; differences between groups are reported as HRs with 95% CIs and were tested using Gray’s test considering competing event analyses. Time 0 reflects the baseline time point when meeting inclusion criteria and admission to hospital. (C) Time-to-event endpoints displayed as Kaplan-Meier curve for the secondary endpoint of heart transplantation or death or transplantation; product-limit survival estimates with number of subjects at risk and 95% Hall-Wellner bands; differences between groups are reported as HRs with 95% CIs and were tested using log-rank tests. Time 0 reflects the baseline time point when meeting inclusion criteria and admission to hospital. (D) Time-to-event endpoints displayed as Kaplan-Meier curve for the secondary endpoint of heart transplantation or death in the subgroup of critically ill patients presenting in severe heart failure (Ross class IV; n = 16); product-limit survival estimates with number of subjects at risk and 95% Hall-Wellner bands; differences between groups are reported as HRs with 95% CIs and were tested using log-rank tests. Time 0 reflects the baseline time point when meeting inclusion criteria and admission to hospital. MEKi = mitogen-activated protein kinase kinase inhibition.

The primary and secondary endpoints are shown in Table 2. Figure 1 depicts Kaplan-Meier time-to-event curves (Figures 1A, 1C, and 1D) and the cumulative incidence function for the MEKi group compared with control subjects (Figure 1B).

**Table 2:** Unadjusted, Adjusted, and Propensity Score Retrospective Cohort Analysis for Primary and Secondary Endpoints

	Total	Standard of Care plus MEKi Group	Standard of Care Only Group (Control)	Model	HR (95% CI)	*P* Value
Cardiac surgery for outflow tract resection or heart transplant or death	32/61 (53)	5/30 (17)	27/31 (87)	Unadjusted	0.093 (0.035-0.245)	<0.001^b^
				Adjusted^a^	0.06 (0.021-0.173)	<0.001
				Propensity as covariate	0.090 (0.033-0.245)	<0.001
Cardiac surgery for outflow tract resection	21/61 (34)	1/30 (3)	20/31 (65)	Unadjusted	0.029 (0.004-0.216)	<0.001^b^^,^^c^
				Adjusted^a^	0.015 (0.002-0.127)	<0.001
				Propensity as covariate	0.034 (0.005-0.262)	<0.001
Heart transplantation or death	13/61 (21)	4/30 (13)	9/31 (29)	Unadjusted	0.421 (0.129-1.376)	0.14^b^
				Adjusted^a^	0.071 (0.013-0.393)	0.002
				Propensity as covariate	0.190 (0.042-0.852)	0.030
Heart transplantation or death in patients with severe heart failure (Ross class IV) at baseline	9/16 (56)	3/10 (30)	6/6 (100)	Unadjusted	0.127 (0.030-0.531)	0.001^b^
				Adjusted^d^	0.091 (0.012-0-697)	0.021
				Propensity as covariate^e^	0.061 (0.006-0.638)	0.020

Values are n/N (%) unless otherwise indicated.

MEKi = mitogen-activated protein kinase kinase inhibition.

a Adjusted for possible confounders as covariates: sex, age at hospital admission, prematurity, Ross class IV, and prior cardiac surgery for outflow tract resection.

b Differences between groups are reported as HRs with 95% CIs and were tested using log-rank tests.

c In competing event analyses, Gray’s test was applied.

d Given the very small sample size of the subgroup of patients with severe heart failure, the analyses were adjusted for sex and age at hospital admission only.

e In the Ross class IV subgroup analysis, the Firth correction was applied in the calculation of the propensity scores.

The primary endpoint (a composite of cardiac surgery for outflow tract resection, heart transplantation, or death) occurred in 32 patients (5 [17%] in the MEKi group vs 27 [87%] control subjects; HR: 0.09; 95% CI: 0.04-0.25; *P* < 0.001). The secondary endpoint of cardiac surgery for outflow tract resection was reached in 21 patients (1 [3%] in the MEKi group vs 20 [65%] control subjects; HR: 0.03; 95% CI: 0.004-0.22; *P* < 0.001). The secondary endpoint of transplantation or death occurred in 13 patients (4 [13%] in the MEKi group vs 9 [29%] control subjects; HR: 0.42; 95% CI: 0.13-1.38; *P* = 0.14) and in 9 patients (3 [30%] in the MEKi group vs 6 [100%] control subjects; HR: 0.13; 95% CI: 0.03-0.53; *P* = 0.001) in the subgroup of critically ill patients presenting in severe heart failure (Ross class IV; n = 16). Generally, the results of the various analyses, namely, unadjusted, multivariable regression adjustment, and propensity score adjusted, are in agreement in that the resulting CIs largely overlap.

**Figure 2:**
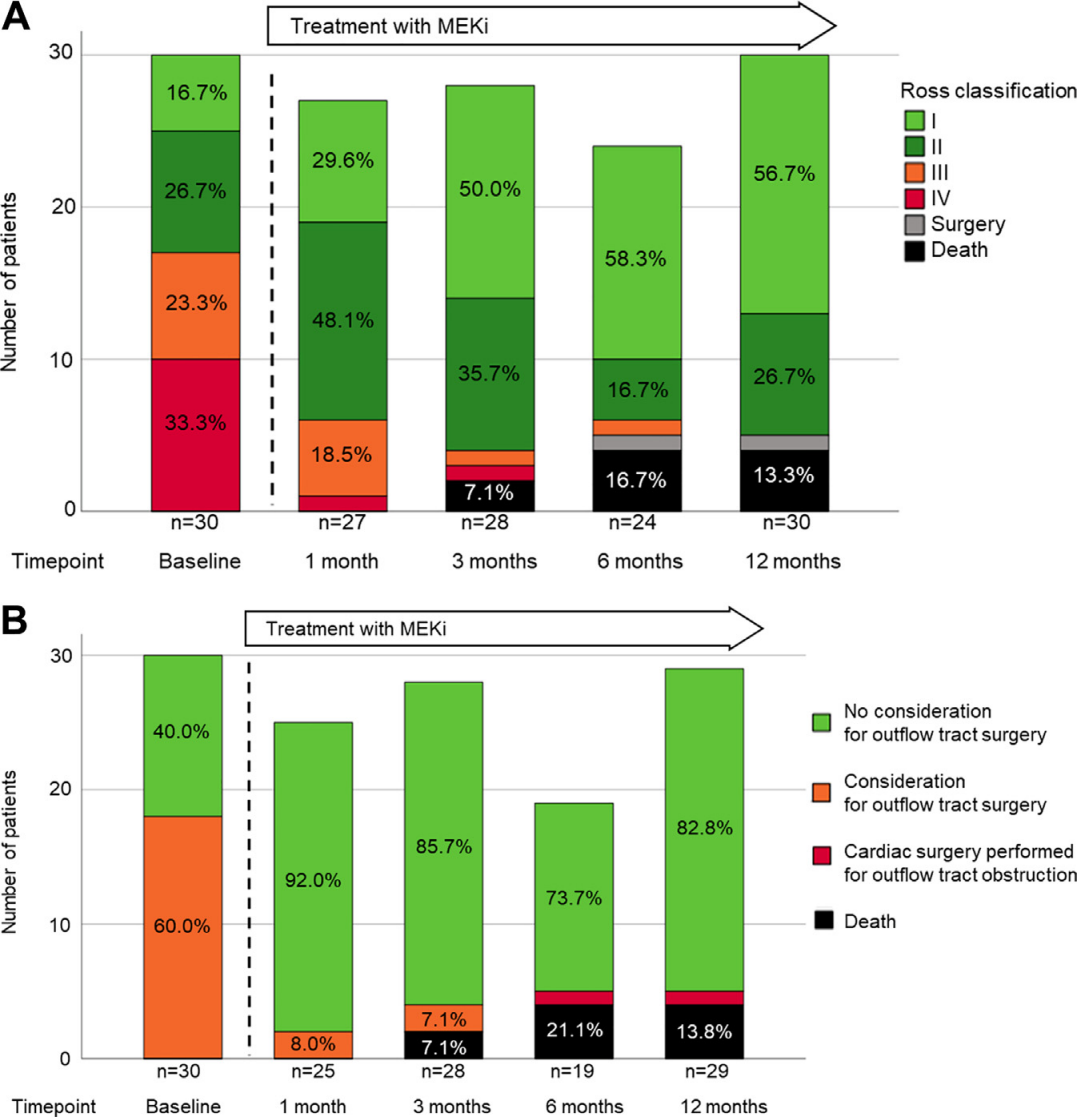
Clinical Outcomes During Follow-Up: Ross Classification and Consideration for Cardiac Surgery for Outflow Tract Resection After the Initiation of MEKi Treatment (A) Ross classification over time in patients in the mitogen-activated protein kinase kinase inhibition (MEKi) group (n = 30). Baseline timepoint reflects clinical status when meeting inclusion criteria and admission to hospital; Pearson chi-square *P* < 0.001. (B) Consideration for cardiac surgery for outflow tract resection according to current guidelines^33^ over time in patients in the MEKi group (n = 30). Baseline timepoint reflects clinical status when meeting inclusion criteria and admission to hospital; Pearson chi-square *P* < 0.001.

The majority of patients in the MEKi group improved in their Ross classification after treatment initiation. The majority of patients, in which cardiac surgery for outflow tract obstruction was considered at baseline according to current guidelines or because of severe outflow tract obstruction, lost their indication after initiation of MEKi (Figure 2).

From baseline to last follow-up, there was greater improvement of Ross classification with decreased need of cardiovascular medications and less myocardial hypertrophy in the MEKi group compared with control subjects (Table 3).

**Table 3:** Clinical Events During Follow-up (Administratively Censored at 36 Months After Baseline)

	Total (N = 61)	Standard of Care Plus MEKi Group (n = 30)	Standard of Care Only Group (Control) (n = 31)	*P* Value^a^
Follow-up time, mo	18 (8.6-36) [0.25-36] (n = 61)	16.5 (11.8-23.5) [3-36] (n = 30)	36.0 (2.9-36.0) [0.25-36] (n = 31)	0.35
Age at last follow-up, mo	45.5 (17-135) [1.25-367] (n = 61)	30 (19.6-67.4) [3-229] (n = 30)	95 (12.0-182) [1.25-367] (n = 31)	0.16
Invasive procedures after the hospital admission				<0.001
None	36 (59)	26 (87)	10 (32)	
Cardiac interventional catheterization	2 (3)	1 (3)	1 (3)	
Cardiac surgery	22 (36)	3 (10)	19 (61)	
Cardiac interventional catheterization and cardiac surgery	1 (2)	0	1 (3)	
Heart transplantation	1 (2)	0	1 (3)	
Surgical outflow tract resection	21 (34)	1 (3)	20 (65)	<0.001
LVOTO resection	18 (30)	0	18 (58)	<0.001
RVOTO resection	8 (13)	1 (3)	7 (23)	0.053
LVOTO and RVOTO resection	5 (8)	0	5 (16)	0.029
Change^b^ in Ross classification	−1 (−2 to −0.5) [−3 to +1] (n = 55)	−1.5 (−2 to −1) [−3 to 0] (n = 29)	−1 (−1 to −0.4) (−2 to +1) (n = 26)	0.011
Change^b^ in number of cardiac medications	0 (−1 to 0) [−4 to +2] (n = 59)	0 (−1 to 0) [−4 to 0] (n = 30)	0 (0-0) [−3 to +2] (n = 29)	0.006
Echocardiographic variables				
Change^b^ in myocardial wall thickness *z*-score^c^	−0.9 (−2.1 to +0.1) [−4.7 to +2.2] (n = 41)	−1.4 (−2.4 to −0.7) [−4.7 to +2.2] (n = 28)	−0.3 (−1.0 to 0.6) [−1.5 to +2.2] (n = 13)	0.009
Change^b^ in peak outflow tract gradient,^d^ mm Hg	−40 (−65 to −12) [−143 to +64] (n = 53)	−47 (−65 to −29) [−143 to 0] (n = 30)	−37 (−74 to 0) [−95 to +64] (n = 23)	0.10

Values are median (Q1-Q3) [range] or n (%). Shown are clinical measures during follow-up. After baseline, the treatment group was started on off-label or compassionate-use treatment with MEKi in addition to standard of care, whereas the control group continued on standard care. Percentages may not total 100 because of rounding.

Abbreviations as in Table 1.

a Pearson chi-square or Fisher exact test for categorical variables and nonparametric Mann-Whitney test for independent continuous variables.

b Difference in measure between last follow-up and baseline time point.

c Maximal myocardial wall thickness *z*-score of the end-diastolic interventricular septum or the end-diastolic left ventricular posterior wall.

d Peak gradient over left or right outflow tract.

**Figure 3:**
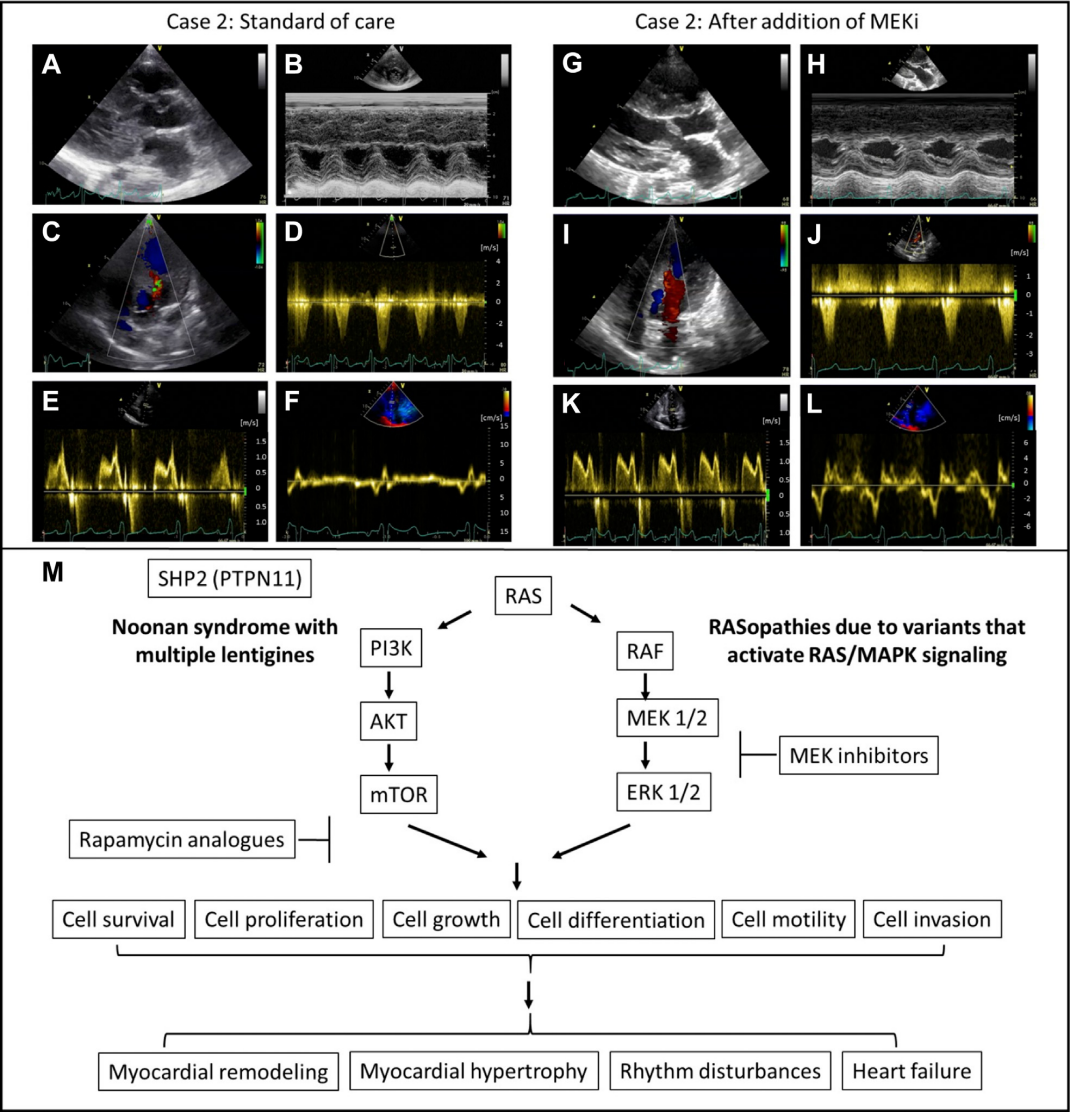
Effect of Treatment on Imaging and Proposed Mechanism of Action Transthoracic echocardiographic measurements of a 8.5-year-old patient carrying a pathogenic *RAF1* mutation before (A to F) and after (G to L) 6 months treatment with the MEK inhibitor trametinib: (A and G): parasternal long axis, (B and H): m-mode; (C and I): apical five-chamber view; (D and J): continuous-wave Doppler through the left ventricular outflow tract on apical five-chamber view; (E and K): pulse-wave Doppler over mitral valve on apical four-chamber view; (F and L): septal pulse wave tissue Doppler on apical four-chamber view; decreased maximal end-diastolic myocardial wall thickness z-score (pretreatment 4.7, A and B; posttreatment 3.2, G and H) and increased end-diastolic left ventricular diameter z-scores (pretreatment −8, A and B; posttreatment −2.7, G and H); decrease in peak left ventricular outflow tract gradient from 88 mm Hg pretreatment (C and D) to 25 mm Hg posttreatment (I and J); improvement of diastolic function variables (pretreatment mitral valve E-to-A ratio 0.7, septal E-to-É 30, E and F; posttreatment mitral valve E-to-A ratio 1.0, septal E/É 20, K and L); (M): hypothesis of mechanism of action of small molecule therapies in patients with RASopathies and cardiomyopathy.

Repeated-measures analysis after the initiation of MEKi showed significant improvement of the laboratory heart failure biomarker NT-proBNP and individual echocardiographic parameters of cardiomyopathy (peak outflow tract gradient, maximal end-diastolic wall thickness *z*-score, left ventricular end-diastolic diameter, and left ventricular mass indexed to body surface area) over time (Supplemental Table 1, Supplemental Figure 3, line plots; example echocardiography in Figure 3).

### Tolerability and duration of MEKi treatment

Forty-nine percent of patients with RAS-CM received the MEK inhibitor trametinib (Novartis) for off-label or compassionate use in addition to standard-of-care treatment at a median age of 5.3 months. Doses of 0.01 to 0.032 mg/kg/d were used. One center performed drug monitoring and confirmed trametinib trough serum levels of 4 to 7 ng/mL achieved with dosing between 0.025 and 0.032 mg/kg/d. MEK inhibitor dosing was adjusted as required by the occurrence of adverse events or on visible treatment effects.

Table 4 summarizes information on MEKi treatment. Median duration of treatment was 16 months (Q1-Q3: 9.5-22.3 months; range: 2-44 months). Treatment was successfully weaned in 5 patients because of sustained improved clinical and cardiac status. Treatment was discontinued in 1 patient because there was insufficient clinical response. In all other living patients, treatment is currently ongoing (20 of 26 living patients). Failure in weaning, defined as worsening of RAS-HCM presenting with an increase in Ross classification, echocardiographic evidence of progression in myocardial hypertrophy and/or outflow tract obstruction, and/or increase in NT-proBNP within weeks of discontinuation or reduction in treatment, occurred in 3 patients. Clinical and echocardiographic improvement was noted at 2 and 4 weeks, respectively, upon resumption of treatment in those patients with weaning failure.

**Table 4:** Information on Compassionate Use and Off-Label Treatment (N = 30)

Dose, mg/kg/d	0.023 (0.019-0.025) [0.01-0.032]
Age at treatment initiation, mo	5.3 (2.0-34.3) [0.5-214]
Duration of treatment, mo	16 (9.5-22.3) [2-44.5]
Ongoing until death	4 (13)
Currently ongoing	20 (67)
Ongoing without weaning attempt	17 (57)
Ongoing after failing weaning attempt	3 (10)
Discontinued because no more indication	5 (17)
Discontinued because no effect	1 (3)
Adverse events	
None	8 (27)
Self-resolving	5 (17)
Requiring symptomatic treatment	10 (33)
Requiring temporary cessation or reduction of treatment	7 (23)
Type of adverse events	
Dermatologic (skin rashes)	13 (43)
Mucous membranes	2 (7)
Gastrointestinal	2 (7)
Mild changes in blood count	0
Mild alopecia	1 (3)
Mild edema	2 (7)
Mild arterial hypertension	1 (3)
Mild hypernatremic dehydration	1 (3)
Mild fingernail irritation	1 (3)
Mild epistaxis	1 (3)
Mild elevation of creatine kinase	1 (3)

Values are median (Q1-Q3) [range] or n (%).

No life-threatening adverse events related to MEKi were noted. Side effects requiring symptomatic treatment occurred in 10 patients (33%). The severity of side effects necessitated temporary cessation and/or dose reduction in 7 patients (23%) (example case in Supplemental Figure 4).

Detailed information on adverse events is listed in Table 4. Cutaneous adverse effects were the most frequent ones observed and were alleviated with topical treatment with emollient cream, topical corticosteroids, and/or topical tacrolimus. Ophthalmologic examinations were performed regularly during MEKi treatment, but no side effects were noted. Additionally, blood counts and serum chemistries (electrolytes and renal, hepatic, and thyroid function tests) were sent regularly for all patients; no abnormalities occurred.

### Description of nonsurvivors in the MEKi group

Three male patients and 1 female patient died during follow-up. The 3 patients had NS due to pathogenic variants in RAF1 (n = 2), RIT1 (n = 1), and LZTR1 (n = 1). Three of the 4 nonsurvivors were born prematurely. All 4 patients met inclusion criteria and were admitted to the hospital for heart failure before the age of 6 months. Three of the 4 patients were in Ross class IV heart failure, and 1 was in Ross class III heart failure. Two of the 4 nonsurvivors had additional pulmonary valve stenosis, 1 had an additional secundum-type atrial defect, 1 had additional aortic valve dysplasia, 1 had mitral valve dysplasia, and 1 had an additional ventricular septal defect. Lymphatic dysplasia was present in 2 and pulmonary hypertension in 2 of the 4 deceased patients. Two of the 4 patients were in respiratory failure requiring mechanical ventilation prior to treatment initiation. Cardiac intervention or cardiac surgery was not performed in any of the nonsurvivors prior to meeting inclusion criteria. Arrhythmias requiring medication were present in 1 of the 4 patients. Two of the 4 patients showed no improvement at all on therapy and passed away as a sequela of progression of disease despite treatment. Two of the nonsurvivors showed improvement of their cardiac status. One of these patients passed away after neurosurgical intervention (placement of a ventriculoperitoneal shunt) for obstructive hydrocephalus as a sequela of prematurity. His cardiac status had been stable prior to undergoing neurosurgery. The other patient passed away from coronary artery ischemia; his cardiomyopathic changes had been stably improved.

## Discussion

This is the first multicenter analysis compiling data on the effect of genotype-dependent, mechanism-based small-molecule therapy targeting the MAPK pathway with comparison with standard of care in a genotyped cohort of patients with RASopathy admitted to the hospital during childhood for severe and life-threatening cardiomyopathy. Our retrospective study corroborates the beneficial effects of MEKi observed in preclinical models^14^^,^^15^ as well as in previously reported anecdotal clinical applications^16^^,^^18^^,^^20^^,^^21^ with data from more patients with other genetic etiologies, longer follow-up, and detailed covariate analysis. The data boost empirical evidence that MEKi is effective in the treatment of selected patients with RAS-HCM carrying genetic variants with activating effects on RAS/MAPK signaling.

On the basis of unadjusted and adjusted retrospective cohort analyses, there were favorable effects of MEKi treatment with regard to the primary composite endpoint of cardiac surgery for outflow tract resection, heart transplantation, or death; the secondary endpoint of cardiac surgery for outflow tract resection; and the secondary endpoint of death or transplantation in the subgroup of the sickest patients presenting with Ross class IV heart failure. After adjusting for covariates including Ross classification at baseline, there was also a favorable effect of MEKi with regard to reaching the secondary endpoint death or transplantation.

The high need for surgical intervention and high mortality of control patients in our cohort of critically ill patients presenting with severe RAS-HCM in Ross class IV were similar to published data from patients with RAS-HCM.^1^^,^^2^^,^^6^^,^^9^ The latter, in particular, is noteworthy: although pediatric cardiac critical care has made important strides over the past couple of decades, the era analysis for this group of critically ill patients with non-NSML RASopathies suggests that outcomes do not appear to have improved significantly.

The direct temporal relationship between the improvement in cardiac variables and the initiation of MEKi treatment as well as the repeated response to MEKi after a worsening due to elective discontinuation of therapy in 3 of the patients suggest that this approach constitutes a viable therapeutic avenue for such cases. Clinical and neurohumoral improvement as well as echocardiographic evidence of reduced outflow tract gradients and myocardial hypertrophy were observed as soon as 4 weeks and lasting until at least 12 months after treatment initiation in most patients (Supplemental Figure 3).

The fact that 2 patients did not tolerate weaning at 10 and 30 months of age but did tolerate it at 39 and 45 months of age may be compatible with age-related variability in the deleterious effects of RASopathy variants on the myocardium in some patients.^5^^,^^9^^,^^35^ This suggests that an optimal treatment window may exist for very young patients with RASopathy, in which MEKi can suppress pathologic activation of the RAS/MAPK cascade and the ensuing cardiomyopathy (Figure 3M). As a consequence, a minimal treatment duration may be required, which still needs to be defined. Of note, treatment was also partially successful in a subset of children presenting with increasing progressive myocardial hypertrophy beyond infancy, suggesting that MEKi may be considered in this group as an alternative to surgical myectomy once conventional medical therapy is exhausted. Interestingly, efficiency of MEKi has also been reported for purely lymphatic indications in patients with RASopathy.^36^^,^^37^

The decision to use MEKi doses lower than those typical for oncological indications was motivated by the fact that RASopathy-associated variants generally engender milder RAS/MAPK hyperactivation than typically observed for cancer-associated variants. Also, RAS-HCM occurs because of a single germline variant, not an accumulation of somatic mutations, potentially within the same pathway. This led us to hypothesize that MEKi trough levels approximately one-half of the typical target for oncologic indication might suffice. We paid particular attention to potential side effects because the susceptibility of patients with RASopathy harboring germline variants to develop novel adverse events was unknown. Our findings, namely, efficacy for low-dose MEKi and reasonable overall tolerance of treatment without occurrence of life-threatening side effects or novel side effects, are compatible with both hypotheses. These observations may help guide future compassionate MEKi use in RASopathies as well as the planning of prospective clinical trials of MEK inhibitors.

### Treatment failures

Two of the 4 patients who died under treatment had severe pulmonary involvement, including respiratory failure and pulmonary hypertension. Two of the 4 patients had concomitant lymphatic dysplasia. Failure to respond to therapy may have resulted from 1 or several of the following causes: RASopathy-associated pulmonary disease may not be as responsive to MEKi treatment, disease processes may have been already too far advanced to respond to MEKi, or other unknown factors. Future efforts should therefore try to delineate comorbidities that contraindicate or limit MEKi therapy.

### Study limitations

First, the conclusions that can be drawn from our experience with MEKi treatment of RAS-CM on the basis of this retrospective data collection clearly do not reach those from a clinical trial with prospectively determined and nearly complete collection of data. There are likely unrecognized biases that need to be accounted for when interpreting our results. Also, our serial data collection was sparser for control subjects than for the treatment cohort. Patients were treated with standard medical therapies as to the treating physicians’ judgement. Minor dosage modifications of concomitant medications used while receiving MEKi treatment might also have influenced disease courses. Also, the subgroup of control group children who was admitted because they needed elective or urgent surgery as to the treating’s physician indication which was not defined by the 2014 guidelines in those admitted before 2014. However, our data show that all those patients met the 2014 guidelines, and hence both clinical status as well as outflow tract gradients at baseline timepoint were similar between groups. Additionally, given the definition of inclusion criteria, there were no patients in the control group who had undergone prior outflow tract surgery, whereas 20% of the MEKi group had undergone prior surgery. This was the only difference in baseline characteristics of the 2 groups, and it was unavoidable because of the design of this retrospective study. However, clinical presentation at the time of meeting inclusion criteria was identical between groups, regardless of prior cardiac surgery. Second, Kaplan-Meier subanalysis including only patients without prior surgery showed similar results as shown in Supplemental Figure 5. Last, the follow-up time in patients of the treatment cohort was shorter than that of the control cohort, albeit not statistically significantly so (Table 3). This was unavoidable given that MEKi treatment was first used in 2017 and that control data were available from patients admitted since 2000. Still, this could potentially lead to an underestimation of the true frequency of side effects or the nonrecognition of adverse effects that are specifically due to long-term treatment. Another confounding factor might be an era effect, given the aforementioned differences in hospital admission date. However, no era effect in morbidity and mortality were observed in a previous national multicenter study nor in the analysis of era among individuals in this study who received standard of care.^1^

The second major limitation is that our data cannot address the question of optimal dosing of MEKi for RAS-HCM. We are mindful that optimal doses may depend on genotype, age, and other factors, and that dose finding will require additional studies in the future.

A third limitation is the length of observation. Although this series provides the longest individual and collective follow-up of RAS-HCM treated with MEKi, we cannot exclude that some patients may develop either resistance to treatment or relapse after cessation of therapy in the long term.

## Conclusions

Retrospective data from this largest international multicenter cohort of rare disease patients with RAS-CM provide evidence of a favorable impact of targeted therapy with MEKi on morbidity and mortality in genotype-selected pediatric patients. Although a minimal optimal treatment window may exist for severe cases, this approach may still be efficient in older children and adolescents doing clinically well but in need for surgical intervention given severe outflow tract gradients.

Taken together, our genotype-specific treatment results establish proof of principle to support the future development of treatment strategies for all RAS-CM, but potentially also for additional RASopathy manifestations. Although still preliminary, these results urgently call for clinical trials to establish indications, long-term efficacy, long-term side effects, surveillance strategies, optimal dosing, and optimal treatment windows.

Perspectives **COMPETENCY IN MEDICAL KNOWLEDGE:** Activating germline mutations in the RAS/MAPK pathway can cause early childhood-onset RAS-HCM, associated with high mortality and morbidity due to heart failure and the need for cardiac surgery, specifically if presenting during early infancy. Preclinical studies in RASopathy mouse models provide evidence that inhibition of the respective hyperactivated pathways leads to disease prevention and amelioration of existing pathology. The use of small-molecule MEK inhibitors might be beneficial in patients carrying activating variants in the RAS/MAPK pathway and in whom standard heart failure therapies are unsuccessful.

**TRANSLATIONAL OUTLOOK 1:** We translated knowledge on the molecular mechanisms of RASopathies into compassionate, off-label use of small-molecule inhibitors of the hyperactivated RAS/MAPK pathway. This retrospective analysis provides the first systematic clinical evidence of the potential of MEKi to reduce mortality and the need for invasive treatment in children with RAS-HCM.

**TRANSLATIONAL OUTLOOK 2:** These findings thus create a more reliable scientific basis for the use of these personalized treatment options in this patient population. The data provided encourage the initiation of clinical trials of these medications and give impetus to widen the search for genotype-specific therapies for these indications.

## Funding Support and Author Disclosures

Dr Wolf is supported by the Gerd-Killian Award of Deutsche Herzstiftung, Deutsches Zentrum für Herz-Kreislauf-Forschung (German Center for Cardiovascular Research) and by Bundesministerium für Bildung und Forschung (German Ministry of Education and Research); and is member of the European Reference Network for Rare and Low Prevalence Complex Diseases of the Heart. Dr Zenker has received funding from Bundesministerium für Bildung und Forschung as part of national and European research networks on RASopathies (German Network for RASopathy Research; FKZ: 01GM1902A) and European Joint Programme on Rare Disease funding (NSEuroNet consortium; FKZ: 01GM1921A). Several authors of this publication are members of the European Reference Network on Rare Congenital Malformations and Rare Intellectual Disability, which is funded by the EU4Health program of the European Union, under grant agreement 101085231 (National Children’s Research Centre). Dr Norrish is supported by the Association for European Paediatric and Congenital Cardiology and Great Ormond Street Hospital Charity. Dr Kaski is supported by a Medical Research Council Clinical Academic Partnership Award and by the British Heart Foundation, Action Medical Research, Max's Foundation, and the Great Ormond Street Hospital Children's Charity. Dr Gelb is supported by a grant from the National Institutes of Health (HL135742). Dr Andelfinger is supported by the Banque Nationale Research Excellence Chair in Cardiovascular Genetics. None of the sponsors or funders had any role in the design and conduct of the study; in the collection, analysis, and interpretation of the data; and in the preparation, review, or approval of the manuscript. Dr Wolf has received honoraria from Novo Nordisk and Bristol Myers Squibb; is a consultant for Bristol Myers Squibb, Rocket Pharmaceuticals, Day One Biopharmaceuticals, BioMarin Pharmaceuticals, Adrenomed, and Pliant Therapeutics. Dr Zenker has received honoraria: Novo Nordisk; and is a consultant or advisory board member for Day One Biopharmaceuticals, Novo Nordisk, and Novartis. Dr Friede has received personal fees from Actimed, Aslan, Bayer, Biosense Webster, Bristol Myers Squibb, CSL Behring, Enanta, Fresenius Kabi, Galapagos, Immunic, IQVIA, Janssen, Johnson & Johnson Medical, KyowaKirin, LivaNova, Minoryx, Novartis, RECARDIO, Recordati, Relaxera, Roche, Servier, Viatris, VICO Therapeutics, and Vifor for statistical consultancies, including data monitoring committees, all outside the submitted work. Dr Kaski has received honoraria from Novo Nordisk; and is a consultant for Novo Nordisk. Dr Gelb is a named inventor on issued patents related to *PTPN11*, *SHOC2*, *RAF1*, and *SOS1* mutations. The Icahn School of Medicine at Mount Sinai licensed the patents to several diagnostics companies (Correlegan, GeneDx, LabCorp, and Prevention Genetics) and has received royalty payments, some of which are distributed to Dr Gelb. Dr Gelb was the recipient of a sponsored research agreement from Onconova Therapeutics; and was a consultant for Day One Biopharmaceuticals and BioMarin Pharmaceuticals. Dr Andelfinger is a consultant for Day One Biopharmaceuticals and BioMarin Pharmaceuticals. All other authors have reported that they have no relationships relevant to the contents of this paper to disclose.
