# Supplementary material for: Impact of MEK Inhibition on Childhood RASopathy-Associated Hypertrophic Cardiomyopathy
Source: JACC Basic Transl Sci. 2024 Dec 4;10(2):152–66. doi: 10.1016/j.jacbts.2024.10.002 (PMC11897442; doi:10.1016/j.jacbts.2024.10.002)
Supplement: Supplemental Material [file mmc1.pdf]

SUPPLEMENTAL APPENDIX

TABLE OF CONTENTS

SUPPLEMENTAL TEXT: Individual clinical case summaries and description of non-survivors

SUPPLEMENTAL TABLE: Repeated measure analysis of the chemical heart failure biomarker NTproBNP age-adjusted zlog and echocardiographic variables in patients of the MEKi group

SUPPLEMENTAL FIGURES:

Supplemental Figure 1: Year of baseline timepoint (hospital admission and meeting all other inclusion criteria)

Supplemental Figure 2: Era analysis of standard of care only group

Supplemental Figure 3: Individual values of the chemical heart failure biomarker NTproBNP age-adjusted zlog and echocardiographic variables in patients of the MEKi group

Supplemental Figure 4: Adverse effects of MEKi treatment on skin

SUPPLEMENTAL TEXT:

Individual clinical case summaries and description of non-survivors

Case 01:

Syndrome (clinical diagnosis): Noonan syndrome

Molecular genetic diagnosis: RAF1, c.770C>T (p.Ser257Leu)

Compassionate / off-label use: MEKi (trametinib)

A male patient presented with respiratory distress syndrome, severe biventricular myocardial hypertrophy, tachycardia and premature ventricular contractions in bigeminy at birth. Prenatal course was complicated by polyhydramnios and macrosomia, caesarian section was performed at 37 weeks of gestation. Birth weight was at the 86th percentile, birth length was at the 4th percentile. The APGAR scores were 7, 9 and 8. The patient was intubated immediately after birth for respiratory insufficiency and cardiac failure. Oral beta-blocker therapy was started but could not be increased as scheduled due to repeated circulatory, respiratory and renal problems. A clinical diagnosis of Noonan syndrome was made and mutation analysis confirmed the diagnosis by showing a typical mutation in the RAF1 gene. At two weeks of life, the patient became hemodynamically unstable in the setting of ventricular tachycardia and was resuscitated. The patient recovered to sinus rhythm after electrical conversion and administration of intravenous amiodarone and beta-blockade. Echocardiography revealed progressive biventricular hypertrophy with small end-diastolic ventricular cavities and no outflow tract obstruction. Laboratory measures revealed markedly elevated B-type natriuretic peptide. Compassionate use of the MEK inhibitor trametinib was started at two weeks of age and titrated up to a dosing of 0.02 mg/kg/day. The patient´s clinical status slowly stabilized. He received mechanical ventilation for the first 4 weeks of life and further non-invasive respiratory support until 12 weeks of life. Amiodarone was discontinued after two weeks and beta-blockers (propranolol) were continued orally up to a dose of 8 mg/kg/day. There was slow improvement of cardiac status with increased cardiac filling volumes and no further progression of myocardial thickening. The patient´s respiratory status improved and he throve slowly. No further life-threatening events occurred and the patients was discharged from the neonatal intensive care unit at 4 months of age. Other organ systems involved were dysplasia of the lymphatic system with generalized lymphedema treated with fat free diet,

poor feeding requiring the placement of a nasogastric tube, macrocephaly, and cryptorchism. Outpatient follow-ups revealed slow amelioration of echocardiographic maximal myocardial wall thickness z-scores and end-diastolic left ventricular diameters. His NTproBNP levels remained stable. The patient showed a positive neurocognitive development without signs of neurological sequelae.

Trametinib treatment was paused twice in this patient. First, trametinib was discontinued after five weeks of treatment because of rising liver enzymes. At this point, the patient also received amiodarone for ventricular arrhythmias. Amiodarone was discontinued and trametinib was restarted 10 days after pausing when the liver enzymes had recovered. No further abnormalities in biochemistry parameters occurred since then. Secondly, trametinib had to be stopped after 9 months of treatment because of severe ulcerative skin erosions on the entire body of the patient (Supplemental Figure 2). This suspected dermatologic adverse effect improved after stopping trametinib and on topical emollients and corticosteroids. While discontinuing trametinib the patient´s cardiac status worsened with increased tachypnea, tachycardia, and excessive sweating. NTproBNP levels increased by almost 5-fold. Trametinib was restarted at a lower dose and clinical status improved within days; NTproBNP levels decreased back to prior discontinuation. The patient´s cardiac status remained stable without significant skin eczema (some dryness controlled with topical emollients) on a dose of 0.016 mg/kg/day.

At the writing of this manuscript, the patient continues on trametinib, follow-up time is 36 months.

Case 02:

Syndrome (clinical diagnosis): Noonan syndrome

Molecular genetic diagnosis: RAF1, c.770C>T (p.Ser257Thr)

Compassionate / off-label use: MEKi (trametinib)

A female patient presented at the age of 6 months with left ventricular obstructive hypertrophy and hemodynamically insignificant atrial septal defects type II with left-to-right shunting. There were no arrhythmias. After uneventful pregnancy and uncomplicated perinatal course, the infant was clinically doing well. A clinical diagnosis of Noonan syndrome was made and mutation analysis confirmed a mutation in the RAF1 gene. The patient was started on beta-blocker therapy and did well for the following months. At 12 months of age there was clinical deterioration in the setting of increasing left ventricular outflow tract gradients. Surgical septal myectomy and patch-closure of the atrial septal defect were performed. Post-operative course was complicated by prolonged intermittent pericardial effusions which were managed conservatively with systemic corticosteroids and colchicine until the age of 3 years. A second surgical septal myectomy with uncomplicated perioperative course was performed at the age of 5.3 years for again increasing left ventricular outflow tract gradients. The patient presented at the age of 8.5 years with worsening clinical functional status deteriorating from age-adjusted Ross classification of II to III, increasing left ventricular outflow tract gradients, severe diastolic dysfunction, and moderate to severe mitral insufficiency. There was hyperdynamic systolic left ventricular function. Disopyramide was added to high-dose beta-blocker therapy without obvious effect. Cardiovascular magnetic resonance tomography confirmed echocardiographic measurements and revealed an increased myocardial mass of 129 g/m^2. There was scarring at the septal myectomy site, mild late gadolinium enhancement and mildly increased interstitial fibrosis as assessed by T1 mapping in the remaining myocardium. No ventricular or supraventricular arrhythmias were noted. Besides short stature and classical Noonan-like facial dysmorphologies, no other organs were involved.

Treatment with high-dose beta-blockade and disopyramide was continued, and the patient was started on the MEK inhibitor trametinib with a dose of 0.013 mg/kg/day. Off-label treatment with

trametinib was chosen as an alternative to a third surgical septal myectomy for dynamic peak left ventricular outflow tract gradients varying between 50 and 90 mm Hg. One week after starting trametinib treatment, echocardiographic peak LVOT gradient was 15 to 25 mm Hg, there was improvement of diastolic dysfunction parameters (mitral valve E to A ratio prior to treatment: 0.75, after treatment: 1), and mitral insufficiency decreased from moderate to minor. NTproBNP levels dropped from 14800 ng/L to 3290 ng/L. Because of significantly improved clinical status and unchanged improved echocardiographic and laboratory measures, the patient was continued on that dosing for 12 months. Weaning off trametinib 12 months after treatment initiation caused rising outflow tract gradients, worsening clinical status, and rising NTproBNP levels, so the previous dose was again continued and is currently ongoing. Follow-up time now is 25 months.

No side effects besides dry skin not requiring any intervention were noted during her treatment.

Case 03:

Syndrome (clinical diagnosis): Noonan syndrome

Molecular genetic diagnosis: RAF1, c.775T>A (p.Ser259Thr)

Compassionate / off-label use: MEKi (trametinib)

A male patient presented with at the age of 2 months in heart failure with severe biventricular myocardial hypertrophy and left ventricular outflow tract obstruction. A clinical diagnosis of Noonan syndrome was made and mutation analysis confirmed a mutation in the RAF1 gene. Prenatal and perinatal course were uncomplicated, the patient was full-term born via cesarean section. The family history was significant for the mother and the sister also affected by Noonan syndrome.

After initial clinical improvement on high-dose beta-blocker and disopyramide the patient received a surgical septal myectomy at the age of 14 months. Perioperative course was uncomplicated and

the patient was discharged on beta-blocker and disopyramide therapy. The patient presented at 7.5 years of age with progressive myocardial hypertrophy causing severe midventricular right outflow tract obstruction with a peak gradient of 178 mm Hg. He was clinically asymptomatic. Maximal end-diastolic interventricular wall thickness z-score were almost 8, there was a small end-diastolic ventricular cavity with a left ventricular end-diastolic diameter z-score of almost minus 10, and there was severe diastolic dysfunction with inverted mitral valve E to A ratio of 0.7 and a septal E to E´ ratio of 24. There was hyperdynamic left ventricular systolic function (ejection fraction 85%). Cardiovascular magnetic resonance tomography confirmed echocardiographic measurements and revealed an increased myocardial mass of 158 g/m². There was scarring at the septal myectomy site, but no further late gadolinium enhancement and no increased interstitial fibrosis as assessed by T1 mapping in the remaining myocardium. No ventricular or supraventricular arrhythmias were noted. Laboratory measures revealed markedly elevated N-terminal prohormone of brain natriuretic peptide (NT-proBNP) levels (16100 ng/L, normal < 100 ng/L).

Besides short stature, classical Noonan-like facial dysmorphologies, astigmatism, and undescended testes requiring orchidopexie earlier in life, no other organs were involved.

Treatment with high-dose beta-blockade and disopyramide was continued, and the patient was started on the MEK inhibitor trametinib with a dose of 0.011 mg/kg/day. One week after starting trametinib treatment, echocardiography showed an increase in end-diastolic left ventricular diameter z-scores, a decreased of right ventricular outflow tract gradients, and improvement of echocardiographic diastolic function parameters (mitral valve pulse-wave Doppler and septal tissue Doppler imaging). The NTproBNP levels decreased accordingly. Four weeks after discharge the patient experienced a COVID19 infection with mild disease course, no hospital admission was required. However, because right ventricular outflow tract gradient increased to 180 mm Hg during this infection, trametinib was titrated up to 0,023 mg/kg/day. He fully recovered from his

COVID19 infection and RVOT gradients decreased again to 110 to 120 mm Hg measured echocardiographically.

No side effects besides dry skin not requiring any intervention were noted during his treatment. The patient was doing clinically well, but given persistent right ventricular outflow tract gradients of about 120 mm Hg, uneventful right ventricular myectomy was performed 6 months after treatment start. The patient recovered well from surgery and was discharged home. Trametinib was continued until 6 months after surgery and was then slowly weaned off given absence of outflow tract obstruction. Follow-up time now is 15 months.

Case 04:

Syndrome (clinical diagnosis): Noonan syndrome

Molecular genetic diagnosis: RAF1

Compassionate / off-label use: MEKi (trametinib)

A full-term born female patient presented within the first months of age with progressive myocardial hypertrophy, left ventricular outflow tract obstruction, atrial septal defect type II, and moderate mitral insufficiency. No arrhythmias were noted. A clinical diagnosis of Noonan syndrome was made and mutation analysis revealed a pathogenic mutation in the RAF1 gene. The patient was started on oral beta-blocker therapy (metoprolol at a dose of 3 mg/kg/day) and surgical septal myectomy, aortic valve reconstruction, and ASD-closure was performed at 3.5 months of age. Plication of the diaphragm was performed at 4 months of age.

Other organ involvement included skeletal dysplasia with shortening of the proximal tubular bones, chondrodysplasia punctata, macrocephaly, and hypothyroidism.

There was progression of myocardial hypertrophy causing re-obstruction of the left ventricular outflow tract and rising NTproBNP levels in the second year of life. In order to avoid a second open-heart surgery, the patient was started on trametinib and titrated to a dose of 0.023 mg/kg/day. A few months after treatment start, there was marked improvement of clinical status, decrease in NTproBNP levels, and a decreased in left ventricular outflow tract gradients.

At this time (follow-up 23 months since treatment initiation) the patient continues on trametinib and there is no more need for a surgical intervention at the left ventricular outflow tract at this point.

No significant side effects except mild skin rash treated with topical emollients was noted.

Case 05:

Syndrome (clinical diagnosis): Noonan syndrome

Molecular genetic diagnosis: RAF1, c.781C>T, p.Pro261Ser

Compassionate / off-label use: MEKi (trametinib)

A female patient presented with progressive biventricular myocardial hypertrophy, dysplastic pulmonary valve, dysplastic mitral valve with abnormal chordae tendineae and bilateral outflow tract obstruction worsening during the first months of life. This baby was born at 38+1 weeks of gestational age. Prenatal findings included an increased nuchal translucency, absent nasal bone, cervical cysts and polyhydramnios.

Biventricular hypertrophy, atrial septal defect type II and moderate mitral valve insufficiency were diagnosed shortly after birth. A clinical diagnosis of Noonan syndrome was made and molecular genetic testing displayed a mutation in the RAF1 gene.

She was initially managed as an outpatient and beta-blocker therapy was started. Admission to the pediatric cardiology unit was first required at 4 months of age for worsening cardiomyopathy and increasing left ventricular outflow tract obstruction. Uncomplicated surgical septal myectomy was performed, as well as pulmonary valve reconstruction and ASD closure. She was discharged home. Four months after surgery progression of left ventricular hypertrophy and increasing left ventricular outflow tract gradients were again noted. Echocardiography confirmed massive left ventricular hypertrophy with maximal end-diastolic wall thickness z-scores of 4,85 and small end-diastolic left ventricular diameter z-scores of -1,26. There was marked diastolic dysfunction with inverted mitral valve E to A ratio on continuous wave Doppler echocardiography. BNP levels were markedly elevated Compassionate / off-label use of trametinib was started at 10 months of age at a dose of 0.0125 mg/kg/day. Clinical improvement with decreased sweating and increased weight gain was noted within the first 3 weeks of treatment. Follow-up visits showed a decrease in myocardial wall thickness, increase of end-diastolic left ventricular volumes, and a decrease over the left ventricular outflow tract gradient from 137 mm Hg prior to treatment to 27 mm Hg 8 weeks after treatment initiation. There was improvement of echocardiographic diastolic function parameters and NTproBNP levels normalized.

Fourteen months after treatment initiation the patient developed neutropenia after Parvo B19 infection. For safety reasons, trametinib was discontinued. Within few weeks, there was worsening of the LVOT obstruction on echocardiography and an increase in NTproBNP levels, so trametinib was re-initiated. LVOT gradients and NTproBNP levels decreased quickly after re-initiation. The neutropenia was diagnosed to be immunologic (antibodies against granulocites were detected, there have been no abnormalities in the bone marrow examination). Neutropenia persists to date, without serious infection (despite regular daycare visits).

At the writing of this report the patient continues on trametinib and is clinically doing well (follow-up since treatment initiation 19 months).

Side effects possible related to the initiation of trametinib included mild arterial hypertension and mild increase in fasting blood lipids.

Case 06:

Syndrome (clinical diagnosis): Cardiofaciocutaneous syndrome

Molecular genetic diagnosis: BRAF, heterozygous c.770A>G; p.Gln257Arg

Compassionate / off-label use: MEKi (trametinib)

The patient is the second child of healthy non-consanguineous Turkish parents. An increased neck fold thickness was noticed and an atrioventricular canal defect was suspected by ultrasound examination prenatally. The boy was born at term by non-elective caesarean section after an otherwise uneventful pregnancy due to cardiotocographic abnormalities without further complications. Clinical examination after birth disclosed increased nuchal lucency, sparse hair, bitemporal narrowing, down-slanted palpebral fissures, hypertelorism, epicanthal folds, a short nose with broad base, and low-set ears. Postnatal echocardiography excluded an atrioventricular canal defect, but showed marked concentric biventricular cardiac hypertrophy. Treatment with propranolol and later spironolactone was started, but cardiac hypertrophy worsened. Swallowing problems necessitated feeding via a gastric tube right from the beginning. At the age of 4 months, the boy is floppy and has made no motor progress. He does not fix persons or objects with his eyes and often lies in an ophistotonic position. Because of myocardial hypertrophy together with increased nuchal lucency, a RASopathy was suspected and exome sequencing revealed a heterozygous mutation c.770A>G in the BRAF gene. Both parents do not carry this mutation. The

patient's overall condition worsened with time and he showed several daily episodes of desaturation in conjunction with pain, agitation, paleness, cold sweat, and fast pulse, potentially reflecting stenocardia. In view of the downhill course, an individual medical treatment with trametinib was proposed to the parents and was agreed by them. Trametinib was started with an oral dose of 0.025mg/kg/day at age 10 weeks and resulted in rapid improvement of his cardiac function and anatomy without side effects except mild facial eczema and edema. At writing of this manuscript the patient continues on trametinib and is in stable clinical condition (follow-up since treatment initiation 24 months).

Case 07:

Syndrome (clinical diagnosis): Noonan syndrome

Molecular genetic diagnosis: RIT1, c.246T>G; p.Phe82Leu

Compassionate / off-label use: MEKi (trametinib)

This case was previously published [17]. In brief, off-label treatment was started in this female at the age of 3 months because of clinical deterioration requiring resuscitation, mechanical ventilation and pleural tubes for drainage of bilateral chylothoraces in the setting of massive biventricular hypertrophy and bilateral ventricular outflow tract obstruction. The patient also had a valvular pulmonary stenosis for which unsuccessful balloon valvuloplasty was performed at the age of 2 months. After starting trametinib, there was marked improvement of the patient's clinical status, echocardiographic parameters, and cardiac biomarkers (NTproBNP). Follow-up time for the initial case report published was 21 months [17]. At 30 months of age, trametinib was discontinued because of a stable cardiac condition. At this point, end-diastolic maximal myocardial wall thickness z-score were 3.4, the end-diastolic left ventricular diameter z-scores were within normal range, and

NTproBNP levels were only mildly elevated at 531 ng/L. After discontinuation of trametinib, NTproBNP increased to 1894 ng/L within 4 weeks. Restarting trametinib caused a decrease of NTproBNP levels to 788 ng/L after 3 weeks, and to 436 ng/L after 8 weeks.

The patient remained on trametinib until 45 months of age, when it was discontinued again. Since then the patient remained in stable clinical condition off trametinib until the writing of this manuscript (total follow-up time 62 months).

Side effects during trametinib treatment included severe, painful stomatitis when the patient received trametinib orally instead of per gastric tube. The stomatitis improved when reducing trametinib dosing. Mild skin eczema was treated with emollient creams and topical corticosteroids.

Case 08:

Syndrome (clinical diagnosis): Noonan syndrome

Molecular genetic diagnosis: RAF1 (c.770C>T; p.Ser257Leu)

Compassionate / off-label use: MEKi (trametinib)

A male patient presented with progression of outflow tract obstruction and heart failure. A clinical diagnosis of Noonan syndrome was made prenatally when increased nuchal translucency, myocardial hypertrophy, and polyhydramnion were noted. Diagnostic amniocentesis did not reveal any abnormalities and genetic testing revealed a pathogenic *RAF1* variant. Severe polyhydramnion required drainage of the excess amniotic fluid (4 liters) at 34 weeks of gestation. The boy was born at 35+5 weeks gestation.

Postnatal echocardiography revealed biventricular hypertrophic cardiomyopathy with right and left sided outflow tract obstruction and enlarged papillary muscles. The right ventricle appeared as

double chambered right ventricle. No arrhythmias were noted. The patient was initially followed clinically. Other organ involvements included feeding problems and failure to thrive.

Due to progression of myocardial hypertrophy, increasing outflow tract gradients, and worsening clinical status the patient started on trametinib at age of 8.5 months of age with a dose of 0.023 mg/kg/day. Within six weeks after starting trametinib, there was clinical improvement including resolved feeding problems. Echocardiographic parameters of hypertrophy improved, but mitral regurgitation worsened over the following months. Patient was readmitted to the hospital 20.5 months after treatment initiation with severe mitral regurgitation and suscipion of rupture of mitral chordae. There was enlarged atria and clinically heart failure. At the same time, patient was tested positive for influenza type B. Patient received operative mitral plasty and his clinical status improved. Follow-up time at writing this report is 22.5 months). Trametinib side effect included mild skin problems that did not require cessation or reduction of treatment.

Case 09:

Syndrome (clinical diagnosis): Noonan syndrome

Molecular genetic diagnosis: RAF1, c.770C>T; p.Ser257Leu

Compassionate / off-label use: MEKi (trametinib)

This female patient presented during the first year of life with severe myocardial hypertrophy, reverse curvature with left ventricular outflow tract obstruction (peak gradient 56 mm Hg) and hyperdynamic LV systolic function. There was also systolic anterior motion of the mitral valve and mild-to-moderate mitral regurgitation. Diastolic dysfunction, severe left atrial enlargement, and a small left ventricular apical aneurysm were also present. Extracardiac features included typical facial features, ptosis, pectus excavatum; pterygium colli; short stature; developmental delays. A

clinical diagnosis of Noonan syndrome confirmed by molecular genetic testing revealing a mutation in the RAF1 gene was done. The patient was initially managed with Verapamil. At 12 months of age the patient was in clinical heart failure and a treatment with trametinib was considered. However, due to lack of insurance approval, the start of trametinib treatment had to be delayed until the age of 20 months. At the time of treatment start, the patient was no longer in clinical congestive heart failure. Trametinib was titrated to a dose of 0.025 mg/kg/day and continued for 4.5 months. There was no evidence of significant improvement on echocardiography and trametinib was discontinued. The patient was switched from Verapamil to Beta-Blocker therapy. After discontinuation of trametinib the patient developed briefly mild signs of heart failure with mild tachypnea which then improved during further follow-up. Follow-up time at writing of this report is 53 months.

Besides mild alopecia, no significant side effects were noted.

Case 10:

Syndrome (clinical diagnosis): Noonan syndrome

Molecular genetic diagnosis: LZTR1, c.742G>A; p.Gly248Arg

Compassionate / off-label use: MEKi (trametinib)

A male full-term infant was diagnosed with Noonan syndrome due to autosomal dominant LZTR1 variant. There was polyhydramnios and increased nuchal translucency on prenatal examinations. Postnatal echocardiography revealed bilateral myocardial hypertrophy with mid-cavitary obstruction and systolic anterior motion of the mitral valve, a large secundum atrial septal defect, moderate posterior malignment ventricular septal defect, and pulmonary valve stenosis.

Other organ involvement included dysmorphic features and progressive respiratory insufficiency initially requiring supplemental oxygen by high-flow nasal cannula and progressing to respiratory failure at 5 months of age requiring intubation and mechanical ventilation. Magnetic resonance lymphangiogram did not show any evidence for lymphatic dysplasia.

Myocardial hypertrophy progressed despite beta-blocker therapy (propranolol 4 mg/kg/day) and trametinib was started at 2 months of age starting with a dose of 0.025 mg/kg/day and titrating up to 0.032 mg/kg/day. Despite treatment, the patient´s clinical status deteriorated and right-to-left shunting through the VSD occurred in presence of severe RVOTO. There was ultimately intractable desaturation and the patient died about 3 months into treatment.

Side effects of trametinib treatment included mild dermatitis.

Case 11:

Syndrome (clinical diagnosis): Noonan syndrome

Molecular genetic diagnosis: RAF1, c.770C>T; p.Ser257Leu

Compassionate / off-label use: MEKi (trametinib)

This case was previously published [19]. A female patient diagnosed with Noonan syndrome secondary to a pathogenic RAF1 mutation presented with moderate cardiac hypertrophy, ectopic atrial tachycardia, atrial septal defect, and valvar pulmonary stenosis at two months of age. Medical therapy with Beta-blockers (Nadolol), furosemide, chlorothiazide, aldosterone antagonism, amiodarone, and verapamil was initiated. The multifocal atrial tachycardia was treated with escalating anti-arrhythmic therapy including amiodarone, flecainide and Beta-blocker (Nadolol). Diltiazem was substituted for flecainide for rate control. Diagnostic cardiac catheterization revealed moderate pulmonary stenosis, no intervention was performed.

Extracardiac features included laryngobronchomalacia, failure to thrive, and developmental delay. Because of multiple cardiac arrests in the setting of unretractable ectopic atrial tachycardia despite maximal anti-arrhythmic management and heart failure, Trametinib was started and titrated to a dose of 0.01875 mg/kg/day at 2 months of age. There was marked improvement of clinical status and tachycardia was controlled within the first days after treatment start. The patient continued to improve and anti-arrhythmic medications were weaned over the following weeks. The patient was ultimately discharged home on beta-blocker and trametinib only. The patient underwent surgical ASD-closure and pulmonary valvotomy 5 months after initiation of therapy. Post-operative course was complicated by post-peridardiotomy syndrome and recurrence of atrial tachycardia. Anti-arrhythmic therapy was again initiated and the patient was discharged home 3 weeks after surgery. Since then the patient has been clinically stable and she continues on trametinib treatment at the time of writing. Follow-up time is 22 months.

Side effects included mild transient diarrhea, which resolved with symptomatic therapy and reduction of trametinib dose by 25%.

Case 12:

Syndrome (clinical diagnosis): Noonan syndrome

Molecular genetic diagnosis: RIT1, c.246T>G; p.Phe82Leu

Compassionate / off-label use: MEKi (trametinib)

This female patient presented after birth with severe pulmonary valve stenosis that progressed quickly within the first month of her life and required a balloon angioplasty. The pulmonary valve annulus was normal in size with thickened and doming leaflets. There was also moderate concentric bilateral ventricular hypertrophy and outflow tract obstruction. She also had moderate mitral valve

regurgitation secondary to poor central coaptation and a small patent foramen ovale with left to right shunting.

Extracardiac features included typical facial characteristics including a broad forehead, hypertelorism, ptosis, a broad nasal bridge, and low set ears. She has also had multiple hemangiomas on her trunk and her feet.

The patient was started on beta-blocker therapy (1-2 mg/kg/day) and trametinib was initiated and titrated to a dose of 0.025 mg/kg/day because of obstructive myocardial hypertrophy.

Her clinical status improved after treatment start with trametinib and her left end-diastolic ventricular diameters increased. Mitral valve regurgitation improved to mild initially, at last follow-up it was moderate. Her left ventricular outflow gradients remained unchanged, but there was improvement on right ventricular outflow gradients. NTproBNP levels did decrease significantly after treatment start.

At writing of the manuscript, the patients continues on trametinib and weaning medication was not attempted so far. Follow-up time is 17 months.

Notable side effects were a bullous impetigo which required inpatient admission to administer intravenous antibiotics. Those skin lesions have now fully resolved.

Case 13:

Syndrome (clinical diagnosis): Noonan syndrome

Molecular genetic diagnosis: RAF1, c.770C>T; p.Ser257Leu

Compassionate / off-label use: MEKi (trametinib)

After preterm delivery at 34+4 weeks of gestational age the diagnosis of Noonan syndrome was made due to typical facial features, chylothorax requiring right-sided pleuritic chest drain in the

neonatal intensive care unit, non-obstructive hydrocephalus (normal MRI brain structurally but delayed maturation of the arachnoid villi suspected as mechanism for hydrocephalus), and undescended testes. Molecular genetic testing confirmed a pathogenic mutation in the RAF1 gene. Prenatal findings included cystic hygroma. Cardiac features were severe obstructive hypertrophic cardiomyopathy with moderate left ventricular outflow tract obstruction, mild right ventricular outflow tract obstruction, mild pulmonary valve dysplasia, moderate secundum atrial septal defect, and mild bi-atrial dilation. Pulmonary involvement was significant for pulmonary lymphangiectasia with chylothoraces requiring drainage and MCT feeding (Monogen). Computer tomography of the thorax was suggestive of lymphangiectasia. The patient also suffered from pyloric stenosis and required pyloromyotomy, and chronic gut hypomotility requiring PEJ placement. Broviac central line placement was done for difficult venous access. Normal hearing assessment.

The patient was initially managed with beta-blocker therapy (propranolol), diuretics (furosemide, spironolactone) and proton pump inhibitor omeprazole. The patient required intubation and invasive ventilation for respiratory insufficiency. Because of severe bilateral myocardial hypertrophy, outflow tract obstruction, heart failure, and persistent lymphatic effusions, Trametinib was started at 6.5 months of age and titrated to a dose of 0.02 mg/kg/day. Within the first weeks after treatment start there was a marked improve in clinical status, decrease of NTproBNP levels, and a reduction in ventricular outflow tract gradients. His respiratory distress resolved, mechanical ventilation was discontinued and the patient was extubated. He appeared developmentally brighter to his parents. During the following months of trametinib treatment there were increased growth velocity, improved gut motility, less respiratory distress (resolution of chronic tachypnoea), less oxygen requirement, improved developmental progress, a more settled child, and no signs for heart failure. At 9 months of age the patient was admitted to the hospital with severe hypernatraemic

dehydration which resolved with symptomatic treatment. Although trametinib was not thought to be the cause for the dehydration episode, trametinib was temporarily suspended as a precaution and re-started at a 25% dose reduction. A few weeks later trametinib was held for moderate to severe acne eruption, which resolved after cessation of trametinib. However, cardiac status worsened while off trametinib (NTproBNP values rising, LVOT gradient increasing to above 50 mm Hg, and worsening clinical status), so trametinib was restarted again at a reduced dose. At the time of writing the patient continues on trametinib (0.01 mg/kg/day) and is in stable clinical cardiac condition. Follow-up time is 17.5 months.

Case 14:

Syndrome (clinical diagnosis): Noonan syndrome

Molecular genetic diagnosis: RAF1, c.770C>T; p.Ser257Leu

Compassionate / off-label use: MEKi (trametinib)

A male neonate born at 34+3 weeks gestation presented with biventricular myocardial hypertrophy, redundant mitral valve leaflets and chordae, thickened 0ic valve leaflets, bilateral lymphatic pleural effusions and ascites after birth.

In utero, diagnoses of absent ductus venosus, severe biventricular hypertrophy, dysplastic but normal functioning atrioventricular valves, cystic hygroma, and polyhydramnios were made. The clinical diagnosis of Noonan syndrome was confirmed postnatally by the molecular genetic finding of a pathogenic mutation in the RAF1 gene.

The patient required mechanical ventilation for respiratory failure immediately after birth and frequent paracentesis for ascites.

Continuous intravenous beta-blocker therapy (Esmolol 150 mcg/kg/min) was initiated due to runs of ectopic atrial tachycardia. Other medications included diuretics and octreotide.

Trametinib was started at 2 weeks of life because of myocardial hypertrophy and lymphatic effusions at a dose of 0.02 mg/kg/day. BNP values decreased and 3 weeks after treatment start no further paracentesis for ascites was required. His heart failure score and his echocardiographic findings remained unchanged. The patient remained ventilator-dependent and he continued to experience runs of ectopic atrial tachycardia.

The patient passed away at 5 months of age due to progression of disease.

No significant side effects on Trametinib treatment were noted.

Case 15:

Syndrome (clinical diagnosis): Noonan syndrome

Molecular genetic diagnosis: RAF1, c.770C>T; p.Ser257Leu

Compassionate / off-label use: MEKi (trametinib)

This case was previously published[22]. This female premature born neonate presented immediately after birth with obstructive hypertrophic cardiomyopathy, severe congestive heart failure and severe pulmonary hypertension. Dilation of the pulmonary artery was also present on echocardiography.

Extracardiac findings included a typical facies, pterigium colli, short and webbed neck, low-set and posteriorily rotated ears, high anterior hairline with wide forehead and narrow temples, mild hypertelorism, downslanting palpebral fissures, broad nose, and a full tip with deeply grooved

philtrum. There were no coagulation anomalies. The neonate also suffered from intraventricular hemorrhage, likely due to prematurity

A clinical diagnosis of Noonan syndrome was suspected at birth and confirmed by molecular genetic testing four weeks later.

The patient required immediate mechanical ventilation including high-frequency oscillation and a trial with iNO, which was ineffective), and she was started on beta-blocker therapy (propranolol up to 3 mg/kg/day), furosemide, and spironolactone.

Due to severe myocardial hypertrophy and heart failure, trametinib was started and titrated to a dose of 0.022 mg/kg/day.

After treatment initiation there was a prompt improvement in clinical conditions, allowing a progressive and rapid withdrawal from inotropes and weaning from mechanical ventilation. NTproBNP levels decreased and generalized edema improved. Echocardiographic findings improved over the next weeks with a decrease of myocardial hypertrophy, outflow tract gradients, and an increase of end-diastolic left ventricular diameters.

At 2.5 months of age, a ventriculoperitoneal shunt was placed After surgery there was a rapid worsening of congestive heart failure and respiratory status. The infant was placed again on mechanical ventilator support. Pulmonary artery dilation, stable until then, increased to 18 mm. The patient passed away from untreatable congestive heart and respiratory failure mostly related to prematurity and related issues (underwent CNS surgery for ventriculoperitoneal shunt placement and subsequently developed severe CHF, consistent with an increasingly growing dilation of pulmonary artery (aneurism) at 3 months of age.

Post-mortem histology showed diffuse blood congestion and alveolar damage, alveolar lumens with exsudate and histiocytes foamy containing hemosiderin, septal fibrosis, thick-walled pulmonary arteries, thickened inter-alveolar septum with proliferation of blood vessels and

capillaries randomly arranged and spaced apart from the alveolar lumen. Histology report was consistent with an aberrant and excessive capillary proliferation and layering resembling pulmonary capillary hemangiomatosis.

Case 16:

Syndrome (clinical diagnosis): Noonan syndrome

Molecular genetic diagnosis: RIT1, c.104G>C; p.Ser35Thr

Compassionate / off-label use: MEKi (trametinib)

This case was previously published [17]. In brief, off-label treatment was started in this female with severe bilateral myocardial hypertrophy and dysplasia of all four valves at the age of 3.5 months because of progression of myocardial hypertrophy despite the use of high-dose propranolol. Marked improvement of the patient's clinical status, echocardiographic parameters, and cardiac biomarkers (NTproBNP) was noted within three months after starting trametinib. Follow-up time for the initial case report published was 17 months [17].

Trametinib was weaned because of a stable cardiac condition at 30 months of age and restarted soon thereafter because of clinical deterioration after weaning (increasing myocardial wall thickness, clinical signs of heart failure). The patient remained on trametinib until 44 months of age, when it was weaned again. Since then the patient remained in stable clinical condition off trametinib until the writing of this manuscript (total follow-up time 62 months).

Side effects during trametinib treatment included skin rash that resolved with topic corticosteroid treatment and loos hair that resolved after weaning.

Case 17:

Syndrome (clinical diagnosis): Noonan syndrome

Molecular genetic diagnosis: RAF1, c.770C>T; p.Ser257Leu

Compassionate / off-label use: MEKi (trametinib)

Left ventricular hypertrophy with mild midventricular obstruction was recognized on fetal echocardiogram, and clinical and genetic confirmation of Noonan syndrome was made shortly after birth. Propranolol was started at 6 weeks, and up-titrated over the first year to a target dose of 5mg/kg/day. The patient was clinically in Ross classification I, but myocardial hypertrophy was progressive and non-sustained ventricular tachycardia was present on holter monitor. At 2 years and 10 months of age, multiple gliomas were detected in the brain stem and the patient required neurosurgical intervention and ventriculo-peritoneal shunt placement for obstructive hydrocephalus.

After his neurological status had stabilized, at 3 years and 11 months of age, Trametinib was started and titrated to a dose of 0.02 mg/kg/day in addition to beta-blocker therapy (propranolol 5 mg/kg/day), Within 3 months of treatment, a decrease of myocardial hypertrophy was noted, the mid-ventricular obstruction diminished, and there was markedly reduced ventricular ectopy. A decrease of left ventricular mass from 56 g/m^2 to 44 g/m^2 was reported on cardiovascular magnetic resonance imaging 6 months after treatment start. In addition, a decrease of glioma size and improvement of vision secondary to decrease of optic nerve impingement were observed during treatment. The parents reported that milestones were achieved more easily since beginning of trametinib treatment.

At the writing of the manuscript the patient remains on trametinib treatment and his follow-up time is 8 months. No significant side events except for transient mild finger nail irritation were observed.

Case 18:

Syndrome (clinical diagnosis): Noonan syndrome

Molecular genetic diagnosis: RIT1, c.280G>A; p.Ala94Thr

Compassionate / off-label use: MEKi (trametinib)

This male infant was born at 37 weeks of gestational age with chylothorax and facial stigmata. Prenatally, polyhydramnios was noted. Pleural effusions were treated with somatostatin and corticosteroids. At 4 months of age, left ventricular hypertrophy and severe left ventricular outflow tract obstruction were noted. The patient was in heart failure. A clinical diagnosis of Noonan syndrome was made and molecular genetic testing revealed a pathogenic variant in the RIT1 gene. The patient was started on beta-blocker (propranolol 3 – 5 mg/kg/day) and diuretic (furosemide 4 mg/kg/day, aldosterone antagonist 1 mg/kg/day) therapy. Pulmonary hypertension was also present. Trametinib was added at 4.5 months of age because of progressive cardiomyopathy and heart failure. No arrhythmias were noted. Over a period of 6 months after treatment initiation, clinical status improved and BNP levels decreased from 981 ng/L to less than 10 ng/L. There was a reduction in peak gradients over the left ventricular outflow tract from 79 mm Hg to 15 mm Hg. Right ventricular pressures measured by echocardiography over tricuspid regurgitation reduced from 55 mm Hg before treatment to 9mm Hg 6 months after treatment. The patient was readmitted to the hospital 9 months after treatment because of a syncope occurring while bottle feeding at home. Echocardiographic evaluation revealed almost absence of left ventricular hypertrophy, no outflow tract gradients, and normal right ventricular pressures. However, there were signs of

ischemia on electrocardiography during crying. Cardiac catheterization showed almost complete occlusion of the left main stem with retrograde perfusion by a large right coronary artery. The circumflex artery was very small, filiforme, with antegrade perfusion. No intervention was performed. The patient developed ST-elevation, bradycardia, respiratory distress, and cardiac arrest after catheterization procedure. Resuscitation was unsuccessful and the patient passed away at 14 months of age.

Side effects of trametinib include epistaxis and cutaneous rash (dryness of the skin), both self-resolving.

Case 19:

Syndrome (clinical diagnosis): Costello syndrome

Molecular genetic diagnosis: HRAS, c38G>A; p.Gly13Asp

Compassionate / off-label use: MEKi (trametinib)

This female infant was born at 31 weeks of gestational age. At birth, low anterior and posterior hairline, low set ears that appeared small and crumped, posteriorly rotated with uplifted earlobes, coarse hair and lanugo, and chest wall and subglottic hemangioma were noted. Bilateral chylothoraces were present at birth. Cardiac defects included coarctation of aorta, small to moderate VSD (doubly committed), and hypertrophic cardiomyopathy causing dynamic left and right ventricular outflow tract obstruction. A clinical diagnosis of Costello syndrome was made and molecular genetic testing revealed a pathogenic variant in the HRAS gene. The patient was started on propranolol , furosemide and spironolactone. Chylothoraces required bilateral chest tube insertion and they eventually resolved on diuretic treatment. Coarctation of aorta was repaired

surgically with resection and end-to-end anastomosis at 8 weeks of age. In the following weeks of life, there was progressive hypertrophic cardiomyopathy with left and right ventricular outflow tract obstruction and the patient continued to be dependent on non-invasive respiratory support (CPAP) and supplemental oxygen. The patient was started on beta-blocker (propranolol 5 - 8 mg/kg/day) and diuretic (furosemide 1.5 mg/kg/day, aldosterone antagonist 5 mg/kg/day) therapy. Trametinib was started and titrated to 0.0116 mg/kg/day as compassionate use at 4 months of age because of progressive cardiomyopathy and heart failure. No arrhythmias were noted. Upon initiation of MEK inhibition the infant was weaned off respiratory support within a week and became much less irritable. Over a period of 4 months after treatment initiation, clinical status improved and BNP levels decreased. There was a reduction in peak gradients over the right ventricular outflow tract from initially 58 mm Hg to 50 mm Hg. Left ventricular outflow tract gradients remained unchanged. No further cardiac intervention was required. The patient was admitted to the hospital from birth on and discharged at 6.5 months of age. Follow-up time at writing of this report was 11 months.

No side effects of trametinib were noted.

Case 20:

Syndrome (clinical diagnosis): Cardiofaciocutaneous syndrome

Molecular genetic diagnosis: BRAF, c.1403T>C; p.Phe468Ser

Compassionate / off-label use: MEKi (trametinib)

This female infant was born premature at 29 weeks gestational age. At birth, distinctive craniofacial appearance (high forehead that narrows at the temples, a short nose, widely spaced eyes (ocular hypertelorism), outside corners of the eyes that point downward (down-slanting palpebral fissures),

droopy eyelids (ptosis), a small chin, and low-set ears), and cutaneous abnormalities (including xerosis, hyperkeratosis, ichthyosis, keratosis pilaris, ulerythema ophryogenes, eczema, pigmented moles, hemangiomas, and palmoplantar hyperkeratosis) were noted. Subsequently, there was also mild-mod hearing loss, hypotonia, pharyngeal collapse leading to apnea-bradycardia-desaturation events, and abdominal distension. A clinical diagnosis of cardiofaciocutaneous syndrome was made and molecular genetic testing revealed a pathogenic variant in the BRAF gene

Cardiac defects included left ventricular concentric hypertrophy and mild right ventricular hypertrophy, mild mitral valve insufficiency, and two small atrial defects with left to right shunting. The patient was treated with diuretics and beta-blockers (dosing 1.5 to 3 mg/kg/day). Compassionate use of trametinib was started at 8 weeks of age because of progressive myocardial hypertrophy and heart failure. Trametinib was dosed at 0.025 mg/kg/day. Within 3 months of treatment, there was significant clinical improvement with weaning from CPAP to nasal cannula, decrease in myocardial wall thickness, decrease of BNP levels, and decrease of initially mildly elevated outflow tract gradients. Beta-blockers were eventually discontinued. At the time of writing the patient is doing well and remains on trametinib treatment (follow-up 10 months since treatment start). No cardiac interventions were required.

No side effects of trametinib were noted.

Case 21:

Syndrome (clinical diagnosis): Noonan syndrome

Molecular genetic diagnosis: RAF1, c. 770C>T; p.Ser257Leu

Compassionate / off-label use: MEKi (trametinib)

This premature born (34 weeks gestational age) female patient with Noonan syndrome carries a clinical (diminished postnatal growth with oral aversion, short stature, mild ptosis, pronounced forehead and other dysmorphic features) and molecular genetic diagnosis of Noonan syndrome due to a genetic variant in the RAF1 gene. Her cardiac defects included hypertrophic cardiomyopathy with left ventricular outflow tract obstruction and dysplastic mitral valve, initially diagnosed at 2.5 months of age. She received left ventricular outflow tract resection and mitral valva plasty for severe mitral regurgitation at the age of 17.5 months. She presented at 30 months of age with increased left ventricular hypertrophy and worsening dynamic mid ventricular cavity obstruction with a peak velocity of up to 4.1 m/sec. Previously, a peak velocity of 2,6 m/sec had been measured. In addition to the existing therapy with propranolol (3mg/kg/day), off-label treatment with trametinib was started to avoid repeat cardiac surgery (dose 0.025 mg/kg/day). Within 6 months of therapy, gradients over the left ventricular outflow tract reduced to below 20 mm Hg and BNP values almost normalized. At time of writing the patient continues on trametinib (follow-up 15.5 months). No cardiac interventions were required. trametinib was held for 2 weeks for dermatitis which then resolved. When trametinib was restarted skin effects were well controlled with topical treatment.

Case 22:

Syndrome (clinical diagnosis): Noonan syndrome

Molecular genetic diagnosis: RAF1, c. 770C>T; p.Ser257Leu

Compassionate / off-label use: MEKi (trametinib)

This premature born (32 weeks gestational age) female patient with Noonan syndrome carries a clinical (facial stimaga, plagiocephaly, short stature, von Willebrand disease, mild pelviectasis

bilateral) and molecular genetic diagnosis of Noonan syndrome due to a genetic variant in the RAF1 gene. She was diagnosed with hypertrophic cardiomyopathy at 2 months of age but was clinically stable on beta-blocker therapy since then (atenolol 1.5 mg/kg/day). She presented at 7 years and 1 month of age with progressive left ventricular hypertrophy and increasing gradient over the left ventricular outflow tract of max 71 mm Hg. On echocardiography, dilated left atrium, systolic anterior motion of the mitral valve leaflet, and moderate mitral insufficiency was noted. BNP values were elevated to a zlog of 4,8, but she clinically had no signs of heart failure. Off-label treatment with trametinib was started to avoid surgical intervention (dose 0.016 mg/kg/day). Four weeks after treatment start, the left ventricular outflow tract gradient was measured at 36 mm Hg, BNP zlog normalized, and her clinical status remained well. At writing of the manuscript she remains on therapy (follow-up time 7 months). No cardiac interventions were required. No side effects of trametinib were noted.

Case 23:

Syndrome (clinical diagnosis): Noonan syndrome

Molecular genetic diagnosis: RAF1, c.785C>T (p.Asn262IIe)

Compassionate / off-label use: MEKi (trametinib)

A clinical diagnosis of Noonan syndrome was made because of typical facial dysmorphism and the presence of bilateral ventricular hypertrophy and left ventricular outflow tract obstruction at one month of age in this female fullterm born patient. Mild dysplasia of the pulmonary valve causing mild stenosis were also present. Beta-blocker therapy (propranolol) was started and titrated up to 6 mg/kg/day. Compassionate use trametinib was started at 3 months of age because of worsening left ventricular outflow tract obstruction, progressive hypertrophy, and rising NTproBNPs.

Clinically the child was in Ross classification II. After start of trametinib, there was a decrease in outflow tract gradients and NTproBNP levels. Clinical status improved. A 3 day hospital stay was required for bradycardia which resolved on reducing beta-blockers to 2 mg/kg/day. The outflow tract gradients remained low despite lowering the beta-blocker dose. No intervention were required since treatment initiation. Trametinib was discontinued after 11 months of treatment given stable clinical appearance. At writing of the manuscript (follow-up 19 months) the patient continues off trametinib therapy and is clinically doing well.

No side effects besides dry skin rash not requiring any intervention were noted during her treatment.

Case 24:

Syndrome (clinical diagnosis): Noonan syndrome

Molecular genetic diagnosis: RIT1, c.170C>G; p.Ala57Gly

Compassionate / off-label use: MEKi (trametinib)

This case was previously published[21]. This fullterm born female patient carried a clinical diagnosis of Noonan syndrome (facial stigmata, mild hypotonia). A pathogenic variant was detected in the RIT1 gene on molecular genetic testing. Biventricular myocardial hypertrophy with right ventricular outflow tract obstruction, and a dysplastic pulmonary, aortic, and mitral valve with a single papillary muscle were detected on echocardiography at 1 months of age. Because of progression of right ventricular outflow tract gradients off-label treatment with trametinib was started to avoid surgical intervention (dose 0.025 mg/kg/day) at 6 months of age. In the following months, her gradients over the right ventricular outflow tract diminished, NTproBNP levels decreased, her clinical status improved, and left ventricular posterior wall thickness z-scores

ameliorated. Balloon valvuloplasty of the dysplastiv pulmonary valve was performed 22 months after treatment initiation. No further cardiac interventions were required. Besides mild skin dryness, no side effects of trametinib were noted. Follow-up time at writing of this report was 44.5 months.

Case 25:

Syndrome (clinical diagnosis): Noonan syndrome

Molecular genetic diagnosis: RAF1, c.770C>T (p.Ser257Thr)

Compassionate / off-label use: MEKi (trametinib)

A male patient with a clinical (Facial features of Noonan syndrome, mild developmental and language delay, recurring otitis media, cryptorchidism, short stature, pectus anomaly, several hyperpigmented skin lesions, hyperkeratosis plantaris) and molecular genetic diagnosis of Noonan syndrome and hypertrophic cardiomyopathy diagnosed at 4 years of life presented with progressive myocardial hypertrophy causing increasing left ventricular outflow gradients. Her clinical status also deteriorated and NTproBNP levels were elevated. Off-label treatment with trametinib was initiated as an alternative to surgical septal myectomy and was titrated to a dose of 0.025 mg/kg/day. Four weeks after treatment initiation, gradients over the LVOT diminished from above 100 mm Hg before treatment to 40 mm Hg, clinical status significantly improved, and NTproBNP levels decreased. There was further improvement over the following weeks. At time of writing of the manuscript the treatment is ongoing and follow-up time is 16 months.

No cardiac interventions were required since treatment initiation.

As temporary decrease in trametinib dosing was required due to skin lesions which resolved after dose reduction. No further side effects occurred.

Case 26:

Syndrome (clinical diagnosis): Noonan syndrome

Molecular genetic diagnosis: RAF1, c.770C>T (p.Ser257Thr)

Compassionate / off-label use: MEKi (trametinib)

A male patient with a clinical (low set, posteriorly rotated ears, short stature, broad neck, unusual chest shape, cryptorchidism (right), proptosis, hypertélorism, low inserted, high-arched palate, wide neck. Supernumerary teeth. Gingivitis and tooth cavities. Orthodontic treatment. Strabismus. Mild scoliosis. Keratosis piliaris. Café au lait lesions right elbow. Cryptorchidy (right). Rectal polyp, rectal prolapse. Attention deficit disorder) and molecular genetic diagnosis of Noonan syndrome and hypertrophic cardiomyopathy had hypertrophic cardiomyopathy diagnosed prenatally. He received infundibular and subaortic myomectomy and ICD implantation at 6 months of age. He presented at 17 years and 10 months of age with progressive myocardial hypertrophy and increasing left ventricular outflow tract obstruction. NTproBNP levels were highly elevated and the patient´s exercise tolerance decreased. Off-label treatment with trametinib was initiated as an alternative to a second surgical septal myectomy and was titrated to a dose of 0.02 mg/kg/day. Four weeks after treatment initiation, gradients over the LVOT diminished from 100 mm Hg before treatment to 46 mm Hg, clinical status improved, and NTproBNP levels decreased. The parents reported that the patient was much more energetic and for the first time in years he was able to run. There was further improvement over the following weeks. At time of writing of the manuscript the treatment is ongoing and follow-up time is 15 months.

No cardiac interventions were required since treatment initiation.

trametinib was briefly stopped one month after initiation of therapy due to severe facial cutaneous acneiform rash which resolved when dose was reduced and Accutane was started. Mild gastrointestinal issues after initiation of therapy resolved spontaneously.

Case 27:

Syndrome (clinical diagnosis): Noonan syndrome

Molecular genetic diagnosis: RAF1, c.770C>T (p.Ser257Thr)

Compassionate / off-label use: MEKi (trametinib)

A female patient with a clinical (macrocephalic, prominence of superficial scalp and forehead vessels, downslanting palpebral fissures, lightly pigmented irises, intermittent alternating exotropia, broad nasal bridge, broad chest with lateral nipple placement, generalized joint hyperextensibility and soft muscles)

and molecular genetic diagnosis of Noonan syndrome presented at 2 months of age with hypertrophic cardiomyopathy. Other cardiac diagnosis included a small to moderate atrial septal defect and mild pulmonary valve stenosis, neither of those requiring intervention. The patient was started on beta-blocker (Propranolol at 3 mg/kg/day). There was progressive obstruction over the left ventricular outflow tract in the following months and clinically worsening signs of heart failure, with elevated NTproBNP levels (10057 pg/L, zlog 4,8). Off-label therapy with trametinib at a dose of 0.015 mg/kg/day was started at 10 months of age when the child was in clinical heart failure classification Ross II and when the maximal gradient over the left ventricular outflow tract was 66 mm Hg, measured by transthoracic echocardiography. One month after initiation of therapy the clinical status improved to Ross I, NTproBNP levels gradually decreased to zlog values of less than 2, and there was reduction of the maximal gradient measured over the left ventricular outflow

tract to 31 to 34 mm Hg. At the time of writing the patient continues on trametinib (follow-up time 15 months) and no interventions were required. Increase of CK levels was noted after initiation of trametinib therapy. This stabilized during follow-up and did not require cessation of treatment.

Case 28:

Syndrome (clinical diagnosis): Costello syndrome

Molecular genetic diagnosis: HRAS, c.34G>T; p.Gly12Cys

Compassionate / off-label use: MEKi (trametinib)

This male infant was born at 29 weeks of gestational age. Patient was diagnosed with respiratory distress syndrome at birth. Initial hyperinsulism resolved spontaneously over the first few weeks of life. Cardiac findings included pulmonary stenosis and progressive hypertrophic cardiomyopathy. A clinical diagnosis of Costello syndrome was made given distinctive facial features and above comorbidities. Molecular genetic testing revealed a pathogenic mutation in the HRAS gene. Patient presented at 2 months of age with severe myocardial hypertrophy and worsening bilateral outflow tract obstruction, despite beta-blocker therapy. Patient had worsening clinical heart failure. Patient was in Ross classification III to IV when trametinib was started as off-label treatment given worsening hypertrophic cardiomyopathy, increasing outflow tract gradients, and worsening clinical status. Trametinib was titrated to 0,02 mg/kg/day, no side effects were noted. Patients clinical status improved significantly to Ross classification I to II, there was a reduction in end-diastolic myocardial wall thickness z-scores, and in outflow tract gradients. Trametinib was discontinued after 4 months of treatment given resolution of symptoms. Patient remained clinically in stable condition, echocardiographic parameters remained stable with mild hypertrophy and outflow tract gradients below 50 mm Hg. Comorbidities included tracheomalacia

and obstructive sleep apnea requiring non-invasive respiratory support within the first few months of life. Follow-up at the time of writing was 9 months since initiation of trametinib.

Case 29:

Syndrome (clinical diagnosis): Noonan syndrome

Molecular genetic diagnosis: RAF1, c.1082G>C; p.Gly361Ala

Compassionate / off-label use: MEKi (trametinib)

A prenatal diagnosis of Noonan syndrome was made in this full term born male infant. Diagnosis of coarctation of the aorta and hypertrophic obstructive cardiomyopathy at birth. Admitted at birth to another academic hospital. At age 2 weeks coarctectomy, at age 7 weeks balloon dilatation re-coarctation (reduction gradient from 53 to 26 mm Hg). Patient also developed pyloric hypertrophy, conventionally treated with duodenal feeding tube. At age 2 months transferred to this tertiary care pediatric intensive care unit to start treatment with trametinib (compassioante use) for progressive hypertrophic cardiomyopathy and left ventricular outflow tract obstruction. At that point patient was in Ross classification III and BNP levels were elevated. After initiation of Trametinib, clinical improvement and reduction of LVOT gradient within 4 to 12 weeks, also reduction of BNP levels. At the time of writing patient continues on trametinib and is doing well. Follow-up time since initiation of therapy is 18 months. Mild transient edema occurred after starting trametinib, but no side effects to therapy requiring treatment were noted.

Case 30:

Syndrome (clinical diagnosis): Noonan syndrome

Molecular genetic diagnosis: RAF1, c.770C>T; p.Ser257Leu

Compassionate / off-label use: MEKi (trametinib)

Trametinib was started in this 17 year and 5 month old adolescent with Noonan syndrome and hypertrophic obstructive cardiomyopathy as off-label therapy as alternative to undergoing surgical myectomy when the left ventricular outflow tract gradient measured 211 mm Hg, despite therapy with beta-blocker and disopyramide. There was no other cardiac involvement. Over the following months after treatment initiation, LVOT gradient diminished to 47 to 68 mm Hg, with variation depending on patient´s compliance taking the medication. Clinically, the patient was always in good condition (Ross classification I). No surgical outflow tract intervention was performed since start of treatment. As side effects to trametinib, there was diarrhea at initiation, resolving after 2 weeks, and transient dermatitis, resolving after 4 months. None of those side effects required intervention. Treatment is ongoing at the time of writing and follow-up time since initiation of therapy is 18 months.

SUPPLEMENTAL TABLE

Supplemental Table: Repeated measure analysis of the chemical heart failure biomarker NTproBNP age-adjusted zlog and echocardiographic variables (peak outflow tract gradient, maximal end-diastolic myocardial wall thickness z-score, end-diastolic left ventricular diameter z-score, and left ventricular mass indexed to body surface area, from baseline (before initiation of MEKi treatment) and at one, three, six and twelve months follow-up in patients of the MEKi group (n=30). Least-square means with standard errors are provided in the table.

**Parameter**		**Change from baseline**			
	**Baseline**	**Month 1**	**Month 3**	**Month 6**	**Month 12**
**NTproBNP* (zlog)**	4.56 (0.34)<br><br>N=21	-2.17 (0.22)<br>p<.0001<br>N=19	-2.28 (0.29)<br>p<.0001<br>N=17	-2.18 (0.38)<br>p<.0001<br>N=16	-2.41 (0.39)<br>p<.0001<br>N=13
**Peak outflow tract gradient§ (mm Hg)**	74.27 (6.23)<br><br>N=30	-26.64 (3.82)<br>p<.0001<br>N=27	-36.55 (5.13)<br>p<.0001<br>N=27	-38.77 (6.51)<br>p<.0001<br>N=16	-47.69 (6.84)<br>p<.0001<br>N=24
**Maximal myocardial wall thickness# (z-score)**	4.72 (0.27)<br><br>N=28	-0.58 (0.20)<br>p=0.0041<br>N=20	-1.15 (0.24)<br>p<.0001<br>N=26	-1.15 (0.29)<br>p=0.0002<br>N=17	-1.32 (0.31)<br>p<.0001<br>N=24

**Left ventricular end-diastolic diameter(z-score)**	-3.45 (0.40)<br><br>N=27	1.05 (0.34)<br>p=0.0028<br>N=15	1.18 (0.40)<br>p=0.0043<br>N=25	1.07 (0.49)<br>p=0.0296<br>N=18	1.74 (0.50)<br>p= 0.0008<br>N=24
**Left ventricular mass indexed to body surface area[¥] (ml/m²)**	121.5 (9.1)<br><br>N=26	-11.61 (5.17)<br>p=0.0278<br>N=19	-29.33 (6.49)<br>p<.0001<br>N=23	-39.83 (8.11)<br>p<.0001<br>N=15	-35.09 (8.62)<br>p=0.0001<br>N=24

*: all but two institutions reported NTproBNP levels; BNP levels reported in those two institutions were converted to equivalent NTproBNP dosing by multiplication with 4 in order to perform statistical analysis

[§]: peak gradient over left or right outflow tract;

[#]: maximal myocardial wall thickness z-score of the end-diastolic interventricular septum or the end-diastolic left ventricular posterior wall

[¥]: left ventricular mass index calculated by Devereux RB et al. [27] normalized to body surface area

SUPPLEMENTAL FIGURES:

Supplemental Figure 1: Year of baseline timepoint (hospital admission and meeting all other inclusion criteria)

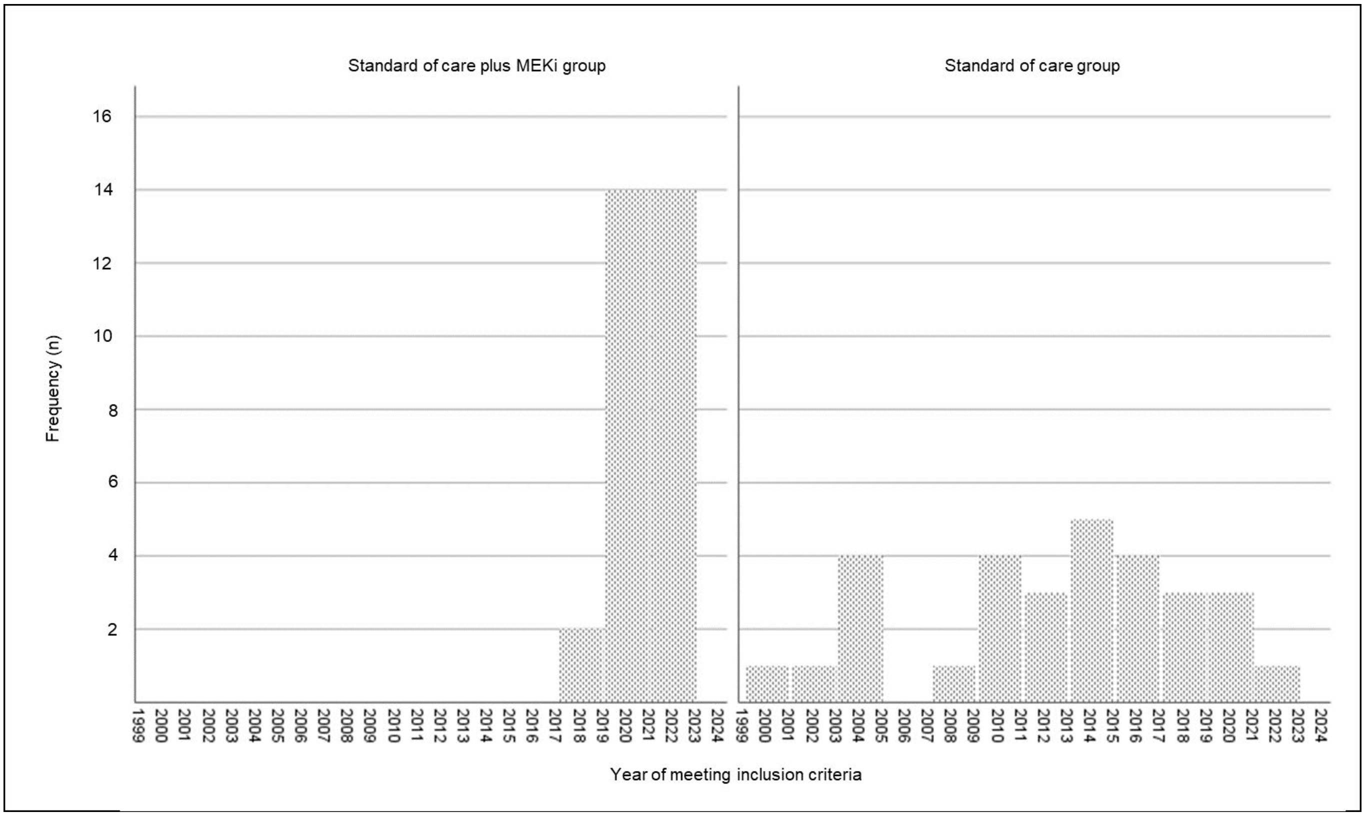

Supplemental Figure 2: Era analysis of standard of care only group

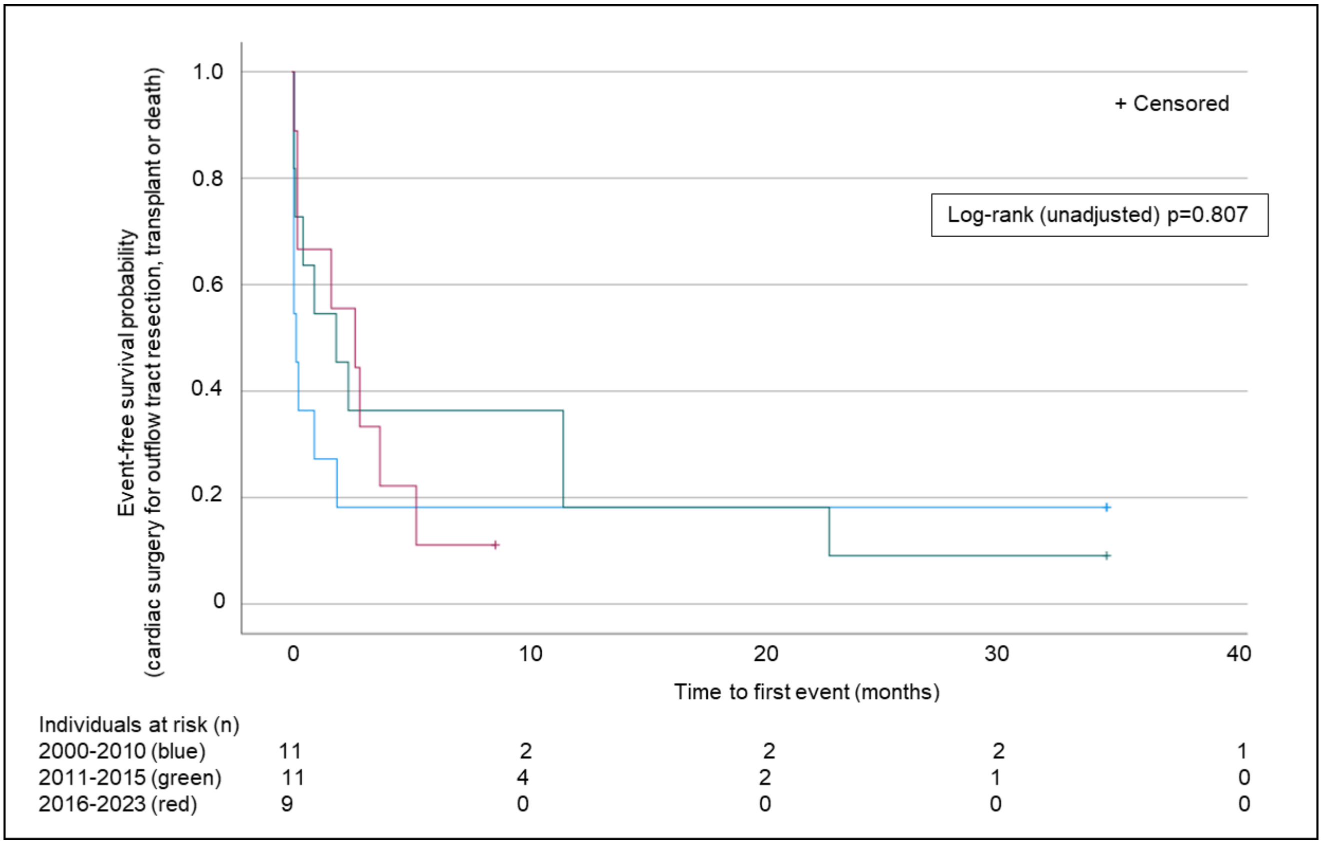

Era analysis of the time-to-event endpoints displayed as Kaplan-Meier curve for primary composite endpoint of cardiac surgery for outflow tract obstruction or heart transplant or death in the standard of care group only; groups reflect year of baseline time point, i.e. hospital admission and meeting all other inclusion criteria between 1999 and 2010 (blue, n=13), 2011 and 2015 (green, n=16), and 2016 and 2023 (red, n=13); product-limit survival estimates with number of subjects at risk; there is no difference in reaching the primary composite outcome between patients admitted in the era 1999 until 2010, 2011 until 2015, and 2016 until 2023. tested by log rank.

Supplemental Figure 3: Repeated measures cardiomyopathic imaging and laboratory measures

Individual values of the chemical heart failure biomarker NTproBNP age-adjusted zlog and echocardiographic variables (peak outflow tract gradient, maximal end-diastolic myocardial wall thickness z-score, end-diastolic left ventricular diameter z-score, and left ventricular mass indexed to body surface area, from baseline (when meeting inclusion criteria and admission to hospital) and at one, three, six and twelve months follow-up in patients of the MEKi group (n=30), with patients experiencing an event censored.

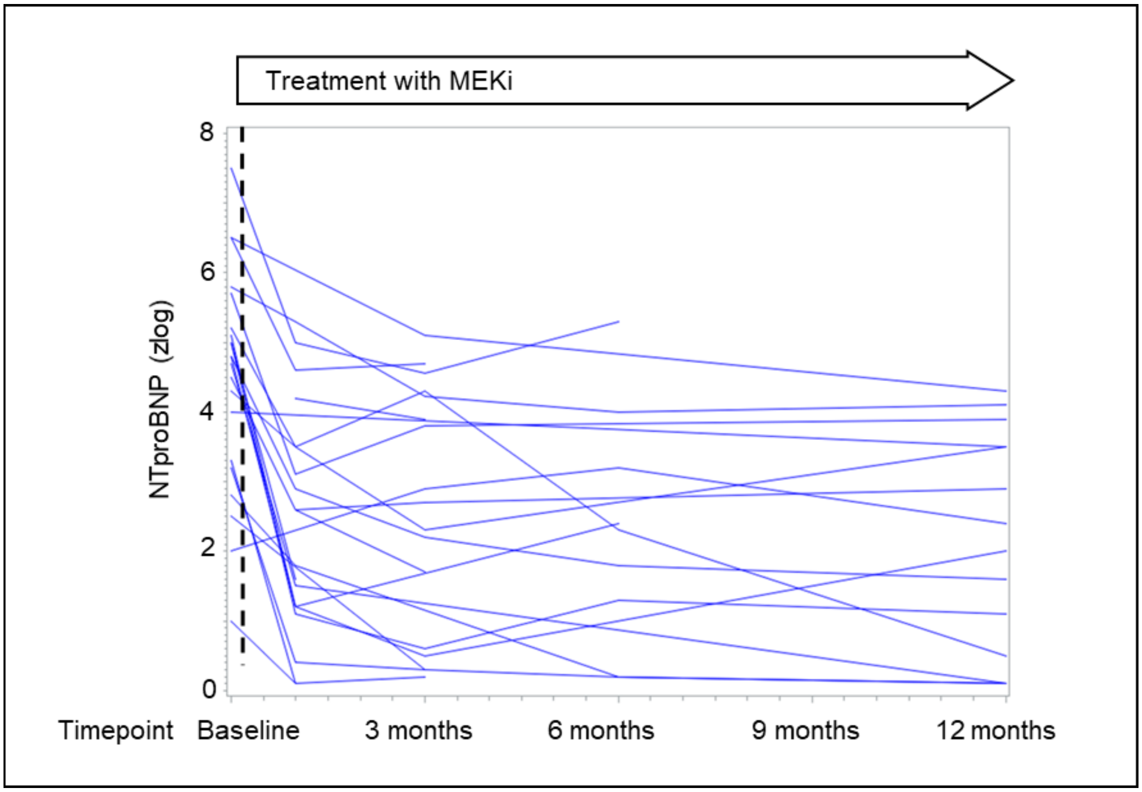

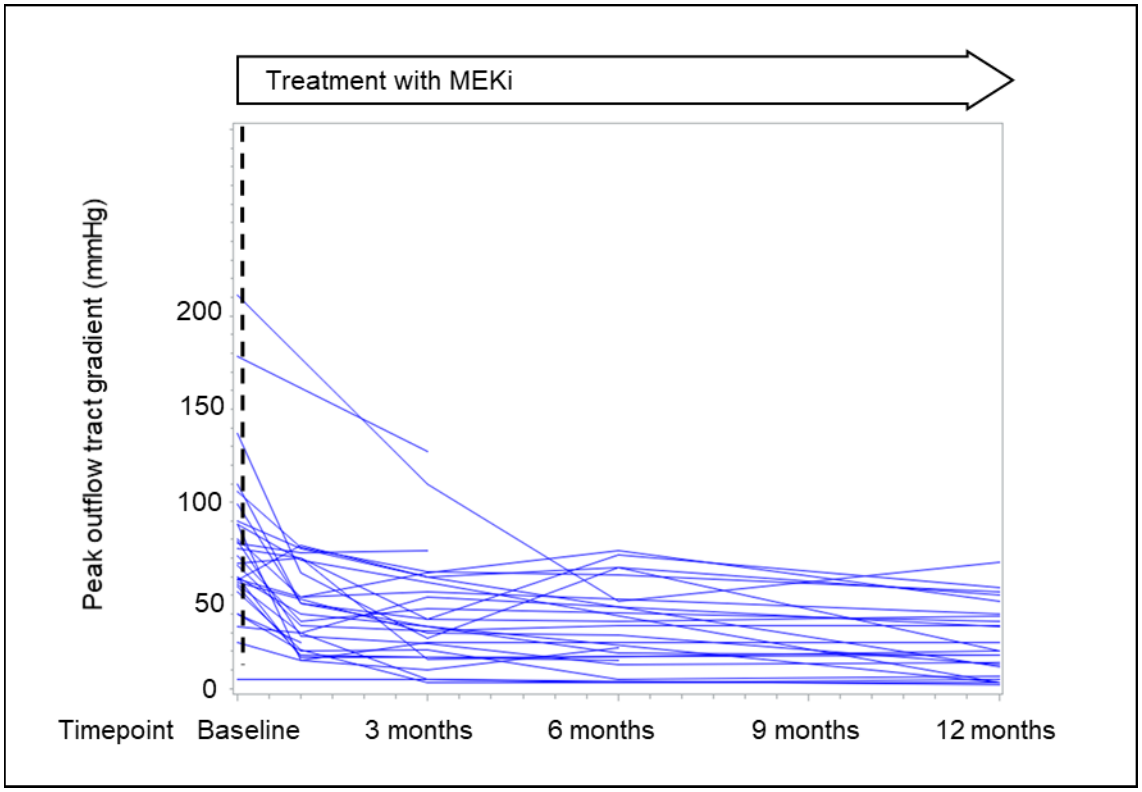
Treatment with MEKi
Peak outflow tract gradient (mmHg)
200
150
100
50
0
Timepoint
Baseline
3 months
6 months
9 months
12 months

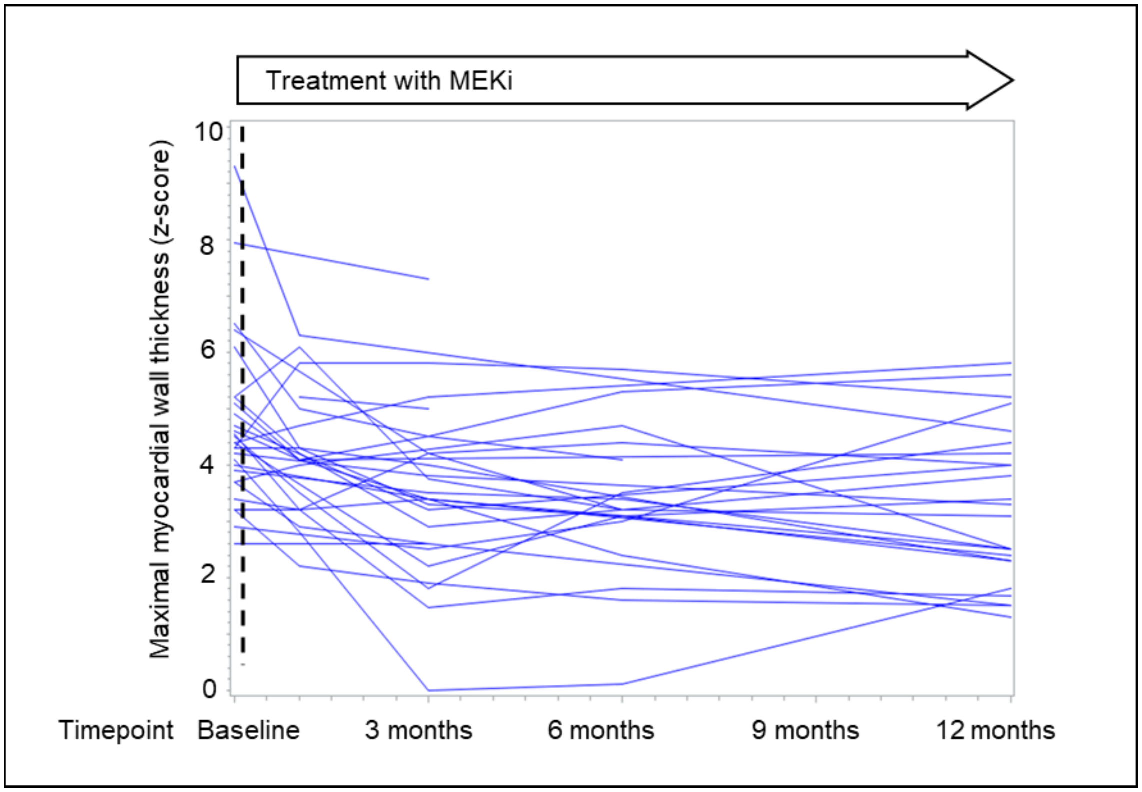
Treatment with MEKi
Maximal myocardial wall thickness (z-score)
10
8
6
4
2
0
Timepoint
Baseline
3 months
6 months
9 months
12 months

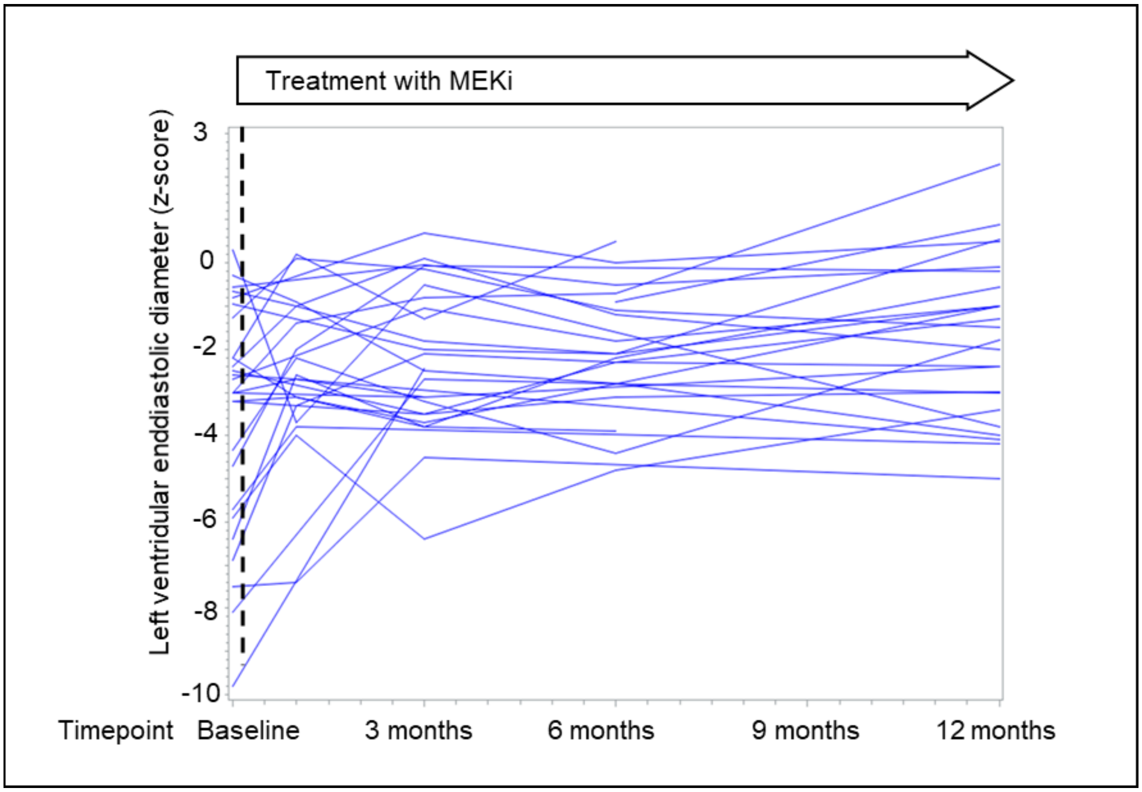
Treatment with MEKi
Left ventridular enddiastolic diameter (z-score)
3
0
-2
-4
-6
-8
-10
Timepoint
Baseline
3 months
6 months
9 months
12 months

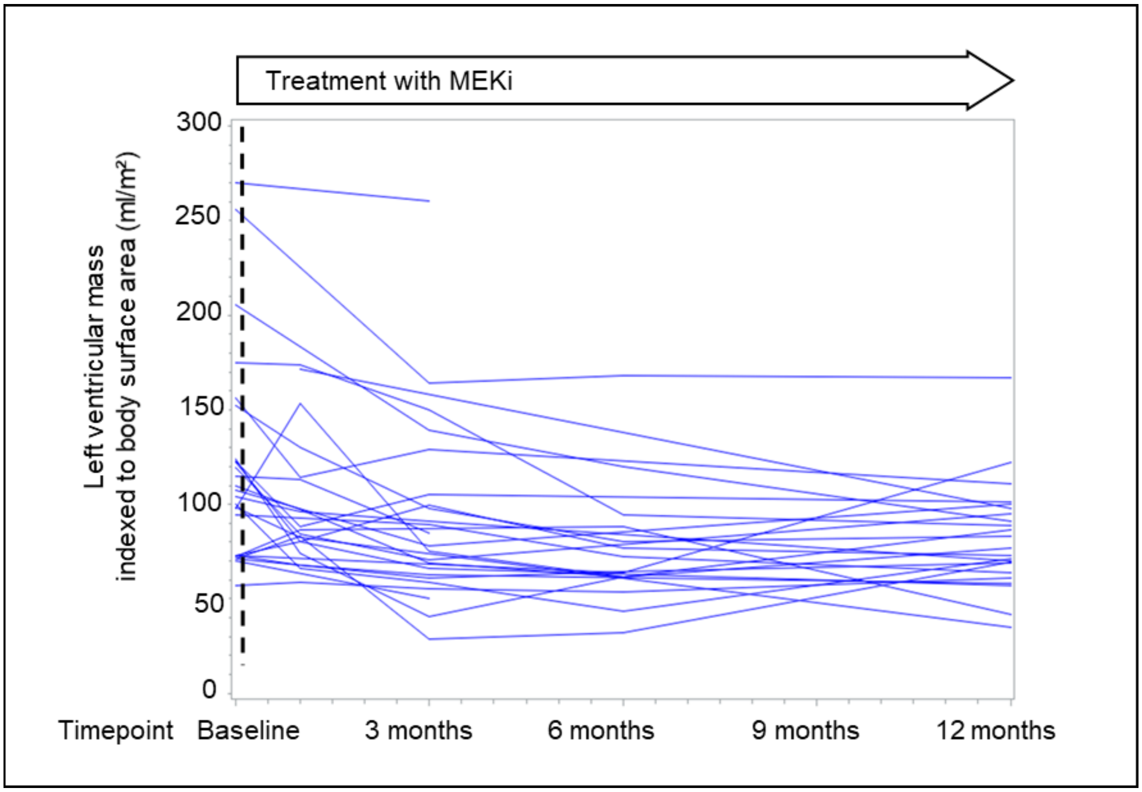
Treatment with MEKi
Left ventricular mass indexed to body surface area (ml/m²)
300
250
200
150
100
50
0
Timepoint
Baseline
3 months
6 months
9 months
12 months

Supplemental Figure 4: Adverse effects of MEKi treatment on skin

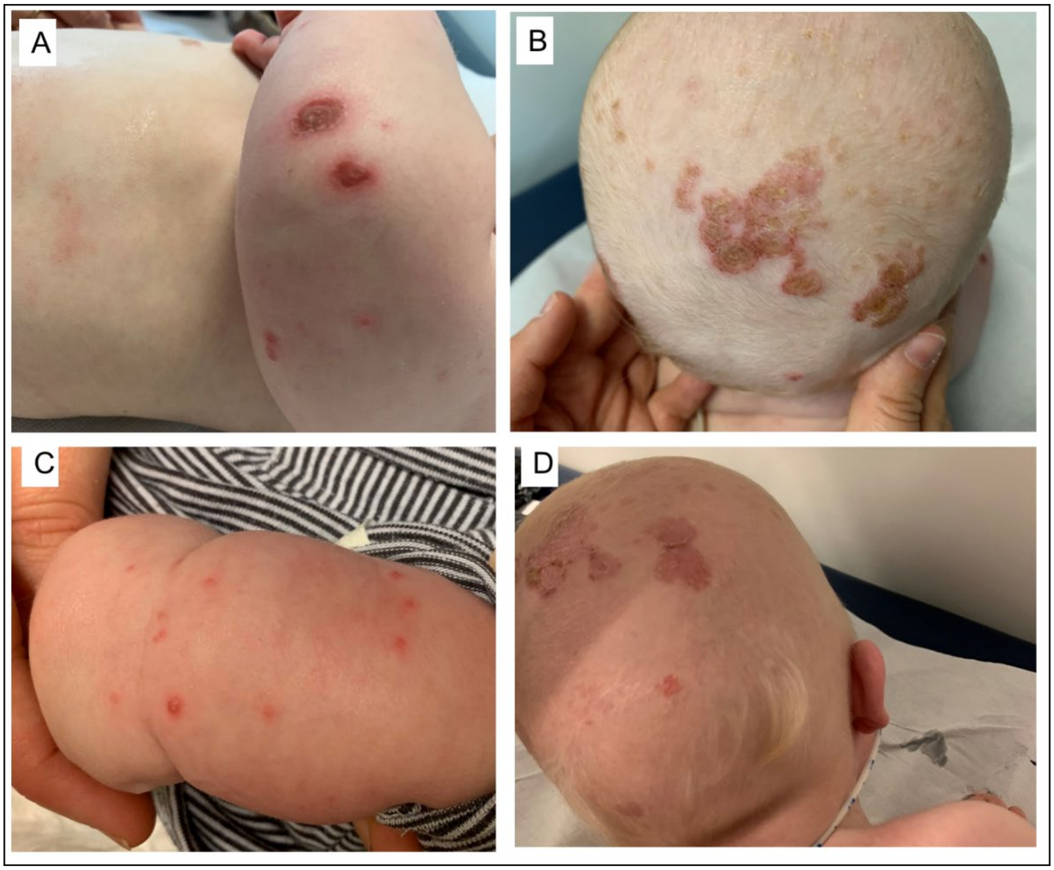

11 month old boy (case 1), pathogenic variant in RAF1, developing an ulcerative rash after 10 months of treatment with trametinib at a dose of 0.015 mg/kg/day (Panel A and B); improvement of rash after cessation of therapy (Panel C and D). As clinical status deteriorated and NTproBNP levels increase within one week of treatment cessation, trametinib was restarting at a decreased dose of 0.01 mg/kg/day. There was improvement in the Ross functional class and a decrease in NTproBNP levels after re-start of treatment; there was no recurrence of adverse cutaneous effects on the lower dose during follow-up (currently treatment ongoing).

Supplemental Figure 5: Kaplan-Meier analysis for primary endpoint in subset of patient without prior cardiac surgery

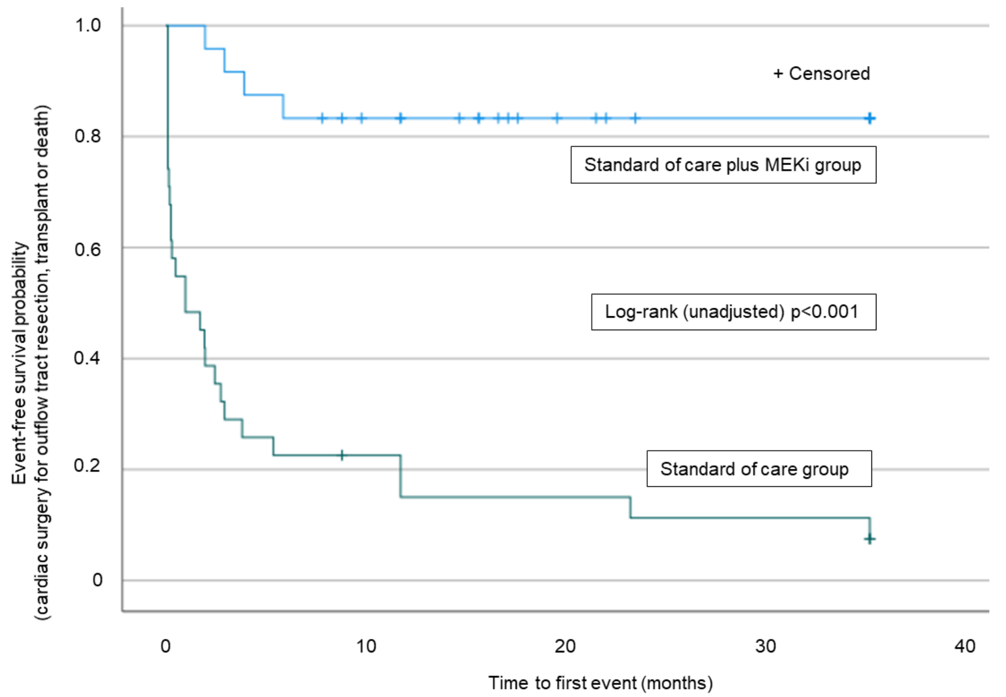

	0	10	20	30	40
Standard of care plus MEKi group (n)	24	18	9	5	0
Standard of case group (n)	31	6	4	3	0
